# Electrochemical Aptasensors for Antibiotics Detection: Recent Achievements and Applications for Monitoring Food Safety

**DOI:** 10.3390/s22103684

**Published:** 2022-05-12

**Authors:** Gennady Evtugyn, Anna Porfireva, George Tsekenis, Veronika Oravczova, Tibor Hianik

**Affiliations:** 1 A.M. Butlerov’ Chemistry Institute, Kazan Federal University, 18 Kremlevskaya Street, 420008 Kazan, Russia; gennady.evtugyn@kpfu.ru (G.E.); porfireva-a@inbox.ru (A.P.); 2 Analytical Chemistry Department, Chemical Technology Institute, Ural Federal University, 19 Mira Street, 620002 Ekaterinburg, Russia; 3 Biomedical Research Foundation, Academy of Athens, 4 Soranou Ephessiou Street, 115 27 Athens, Greece; gtsekenis@bioacademy.gr; 4 Department of Nuclear Physics and Biophysics, Comenius University, Mlynska Dolina F1, 842 48 Bratislava, Slovakia; veronika.oravczova@fmph.uniba.sk

**Keywords:** antibiotics, electrochemical sensors, DNA aptamers, computer simulations

## Abstract

Antibiotics are often used in human and veterinary medicine for the treatment of bacterial diseases. However, extensive use of antibiotics in agriculture can result in the contamination of common food staples such as milk. Consumption of contaminated products can cause serious illness and a rise in antibiotic resistance. Conventional methods of antibiotics detection such are microbiological assays chromatographic and mass spectroscopy methods are sensitive; however, they require qualified personnel, expensive instruments, and sample pretreatment. Biosensor technology can overcome these drawbacks. This review is focused on the recent achievements in the electrochemical biosensors based on nucleic acid aptamers for antibiotic detection. A brief explanation of conventional methods of antibiotic detection is also provided. The methods of the aptamer selection are explained, together with the approach used for the improvement of aptamer affinity by post-SELEX modification and computer modeling. The substantial focus of this review is on the explanation of the principles of the electrochemical detection of antibiotics by aptasensors and on recent achievements in the development of electrochemical aptasensors. The current trends and problems in practical applications of aptasensors are also discussed.

## 1. Introduction

Antibiotics, compounds of natural or synthetic origin with antimicrobial activity, are widely used in human and veterinary medicine. Antibiotics are also added to some products as preservatives due to lack of refrigeration. Rather high amounts of antibiotics are also applied in the livestock industry and aquaculture. The overuse or misuse of antibiotics can result in their accumulation in animal meat and milk. Further use and consumption of food contaminated by antibiotics can lead to antibiotic resistance or allergic reactions, which represents a great threat to humans [1]. In terms of milk products, the presence of antibiotics can, for instance, have a negative impact on the production of cheese, as it inhibits the growth of useful bacteria. It is, therefore, highly desirable to develop fast, sensitive, selective, low-cost, and easy-to-use methods for the detection of antibiotics in food. Currently used assays are based on microbiological methods, as well as on traditional analytical techniques, such as gas chromatography (GC), high-performance liquid chromatography (HPLC), and mass spectroscopy (MS). Higher accuracy of detection can be achieved by a combination of MS with HPLC (MS-HPLC) or GC (MS-GC) [2,3]. Microbiological methods are effective and relatively fast but require sterile, specialized laboratories. Other analytical methods are laborious, require rather expensive instruments, and qualified staff, and usually need sample pretreatment. In addition, HPLC requires organic solvents that pose serious hazards to the environment. The above-mentioned methods are used as standard in food and agriculture laboratories. Therefore, in Section 2, the principles of these methods are explained, and relevant references are provided. Biosensor technology, on the other hand, is a rapidly growing field that offers a promising tool for antibiotics detection [4]. A common attribute among all biosensors is that they consist of recognition elements (receptors) such as antibodies, enzymes, peptides, lectins, nucleic acid aptamers, cells, or synthetic receptors, for example, molecularly imprinted polymers (MIPs) or calixarenes. Receptors are immobilized at the surface of a transducer that converts the affinity or chemical interactions with the target analyte into a measured physical signal such as electrical, optical, or mass change [5,6]. Among the different types of receptors, antibodies and enzymes are widely used in the preparation of biosensors for antibiotics detection [7]. However, the current trend in biosensor design is the application of nucleic acid aptamers for this purpose. This is due to their high sensitivity, selectivity, higher stability in comparison with antibodies, and easy chemical modification that allows their immobilization on various surfaces [8]. The number of articles focused on the development of aptamer-based biosensors (aptasensors) is rapidly growing [4]. Currently, many aptamer sequences have already been selected for various antibiotics using combinatorial chemistry known as “systematic evolution of ligands by exponential enrichment” (SELEX) and by various modifications of this method [8]. Despite the improvements introduced to SELEX and post-SELEX modifications that have allowed the development of aptamers with high affinity for their target analytes, there is still great room for improvement. Therefore, we also include Section 3.2, dealing with the improvement of aptamer binding properties through computer modeling. 

The focus of this review is on recent achievements in the development of electrochemical aptasensors for antibiotics detection. Electrochemical methods, such as voltammetry, amperometry, and electrochemical impedance spectroscopy (EIS), are of great advantage due to their low cost, high sensitivity, and the possibility to detect antibiotics even in non-transparent liquids, which is difficult for optical methods. Therefore, in Section 4, a brief explanation of the principles of the most often used electrochemical methods is presented, together with the method of preparation of aptasensors. The latter includes proper immobilization of aptamers at various surfaces, including nanomaterials such as carbon nanotubes, graphene oxide, or nanoparticles. Therefore, in Section 4.1, an overview of the methods used for aptamer immobilization is provided. Section 5 includes a description of recent achievements in the development of electrochemical aptasensors, as well as a discussion on current trends in aptasensors design. The difficulties in the application of electrochemical aptasensors for the analysis of food contamination are also discussed.

## 2. Conventional Methods of Antibiotic Detection and Food Contamination Limits

Antibiotic resistance occurs when bacteria are exposed to sublethal concentrations of them in the environment. The concentration of antibiotics promoting resistance can be as low as ng/mL [9,10,11,12]. Therefore, it is very important to establish the maximum residue limit of antibiotics in food (MRL). The sensitivity of the analytical methods for antibiotic detection should, therefore, be below MRL. As an example, Table 1 contains the values of MRL for various antibiotics in cow’s milk that were established in the European Union. Usually, the MRL in cow’s milk is lower than the MRL in other food products. 

To prevent the consumption of food contaminated by hazardous compounds, including antibiotics, various detection techniques can be applied. Among conventional methods, microbiological, physical, and chemical assays are most often used. The principles of these methods are briefly explained below.

### 2.1. Microbiological Assays

These assays are based on the evaluation of the effects of antibiotics contained in food samples on bacteria growth. For this purpose, the disc diffusion method is often used in routine analyses in food laboratories. In this method, the disc soaked by a sample of interest, such as milk or homogenized meat products, is placed on top of agar plates containing the bacteria. The presence of antibiotics is evaluated based on bacterial growth around the disc. In order to identify the antibiotic, various bacterial species are used, such as *Bacillus stearothermophilus*. This method has been optimized, particularly for the analysis of milk samples with limits of detection (LODs) between 30 and 80 ng/mL for β-lactam antibiotics, as an example [15,16,17].

Another method used by food laboratories is the colorimetric method. It is based on measurements of the absorbance of the metabolism of certain species of bacteria in the presence of antibiotics. For example, Kalunke et al. [18] reported the detection of ciprofloxacin in cow’s milk, a compound widely used for the treatment of bacterial infection in cattle. For this purpose, they monitored the proliferation of *Escherichia coli* ATCC 11303 in milk contaminated by ciprofloxacin based on the colorimetric assay of β-galactosidase (β-gal) activity in the presence of chromogenic β-gal substrate. The proliferation of *E. coli* has been proportional to the β-gal activity, while it decreased in the presence of ciprofloxacin. The sensitivity of this assay corresponded to 1 MRL, which is 0.1 mg/kg.

Commercial colorimetric kits for antibiotics detection are also available. Le Breton et al. [19] compared the sensitivity of Delvotest^®^ and Copan Milk Test for the detection of various antibiotics in milk such as penicillin G, cloxacillin, sulfamethazine, sulfadiazine, oxytetracycline, gentamicin, cephalexin, cefquinome, dihydrostreptomycin, and trimethoprim. These assays use agar microplates containing *Bacillus stearothermophilus.* The addition of milk induces the production of carbonic acid, which causes changes in the color of the bromocresol purple indicator from purple to yellow. The presence of antibiotics in the sample inhibits the growth of bacteria and the color remains purple. The assay allowed the detection of most antibiotics at a level below MRL by visual inspection of color changes after 3 h of incubation. Similar sensitivity was demonstrated by Delvotest^®^, which is working on the same principles as the above-mentioned commercial tests [15,20]. Colorimetric methods are rather fast (around 3 h); however, as already mentioned, they should be performed in a sterile microbial laboratory.

Microfluidics and paper-based microbiological assays can also be used for antibiotic detection [21,22]. The principle of microfluidic analysis consists of the incubation of the bacteria in the microchannels and monitoring the optical density, fluorescence, or morphological changes upon incubation with antibiotics. For example, Sun et al. [21] used a 7-channel microfluidic device containing 32 square-shaped microchambers that were filled with recombinant *E. coli* HB101 pGLO. Owing to the pGLO plasmid, the bacteria in the presence of L-arabinose produced green fluorescent protein (GFP). Incubation of bacteria with antibiotics tetracycline or erythromycin caused suppression of the bacterial growth and, as a result, a decrease in fluorescence intensity monitored by fluorescence microscopy. In addition, at relatively high concentrations of tetracycline (3 μg/mL), the formation of long filamentous bacteria with a length of approx. 50 μm was observed. This assay allowed detection of the antibiotics, with a sensitivity of around 1 μg/mL.

Deiss et al. [22] reported a portable paper-based assay for the detection of the effect of antibiotics on bacteria. In this assay, the wax-patterned paper had zones for deposition of media (M-zone), a culture zone (C-zone) for bacteria deposition, and two zones for deposition of antibiotics (A-zone). The *E. coli* K12 ER2738 strain, susceptible to antibiotics, was deposited on a C-zone in the amount of 10^5^ CFU (colony forming units). Tetracycline, ampicillin, or kanamycin were deposited onto the A-zones. The bacteria were visible (pink color) due to the addition of PrestoBlue™ (resazurin). Antibiotics resulted in the depression of bacterial growth, which was indicated by changes in color from pink to blue around A-zones. The blue-colored area was used as an indicator of antibiotic concentration. The images were analyzed by a smartphone using image acquisition software for Android platforms. This test can be performed in normal (not sterile) conditions and even by inexperienced personnel. Its only disadvantage is that it requires approximately 18 h of incubation, and it is not specific. This assay cannot distinguish between various antibiotic species. At the same time, knowledge about the concrete presence of different types of antibiotics is important in searching for the source of contamination [23]. 

### 2.2. Chromatographic Methods of Antibiotic Detection

Chromatography is an analytical technique based on the separation of the components in a sample using their partitioning between the mobile and stationary phases. Chromatography is widely used in analytical chemistry and food analysis. It can be subdivided into several types, such as gas chromatography (GC), thin-layer chromatography (TLC), and high-performance liquid chromatography (HPLC). Equally sensitive is also mass spectroscopy (MS). The combination of gas and liquid chromatography with mass spectroscopy (GC–MS or LC–MS, respectively) increases the sensitivity and specificity of detection.

Gas chromatography separates volatile compounds in a gaseous phase. The substance of interest should be transformed into the gaseous phase by heating while the liquid is adsorbed at the solid support. The mobile phase is usually helium or argon. The components of the sample are distributed between the mobile gaseous phase and the stationary phase through which the gas is moved. Due to different properties of the components such as size and affinity in the stationary phase, they will be separated and analyzed via the spectroscopy method, which results in several chromatographic peaks. GC can be applied for the detection of antibiotics in food samples. However, not all antibiotics can be transformed into a gaseous phase. In order to increase the sensitivity of GC, its combination with the electron-capture technique has been used. In this case, the electron-captured carrier gas flows through an electron-captured detector consisting of an anode and a cathode. Some of the electrons of the molecule are captured, and this is recorded as a peak current [24]. This increases the sensitivity of detection. For example, GC in combination with the electron-capture technique allowed detection of chloramphenicol with LOD of 1–10 ng/g in various food samples with a rate of recovery ranging from 72% (unpolished rise) to >90% (milk and meat) [25].

The problems associated with the necessity to transform the sample containing antibiotics into a gaseous phase can be solved by HPLC. The principle is similar to GC, but the mobile phase is liquid. HPLC combines high separation capability with high sensitivity, which is typically below MRL [26,27]. However, this method, despite having high sensitivity and selectivity, requires relatively expensive instruments, qualified staff, and organic solvents. 

Mass spectroscopy is also a rather powerful analytical technique suitable for food analysis. This method identifies molecules based on measurements of their mass to charge ratio, *m*/*z*. This is especially useful for the detection of ultralow quantities of molecules down to 1 pg. The mass spectrometer consists of a sample injector, sample ionizer, mass analyzer, and ion detector. The sample is injected into the ionizer, which ionizes the molecules, and the resulting ions are then detected. The collision of the ions with the gas is prevented with measurements in the vacuum. The resolution of the mass spectrometer is defined as *R* = *m*/Δ*m*, where *m* is the mass, and Δ*m* is the mass difference between two resolvable peaks. This value is typically in the range of 10^2^–5 × 10^5^. The types of MS spectrometers and principles of their operation can be found in Nölting [28], in which a detailed description of the combined techniques of GS–MS and LC–MS can also be found. For example, in LC–MS, the sample is injected into an HPLC separation column. The splitter at the end of this column is used for the insertion of the sample into MS. The resulting two-dimensional diagram allows the sensitive detection of molecules in a complex sample such as cell extract. If the resolution of the chromatographic technique is 10^2^, and that of MS is 10^4^, their combined resolution will be 10^6^. MS is a powerful tool for antibiotic detection, with a resolution well below MRL. However, mass spectrometers, too, are rather expensive instruments and, similarly to the chromatographic methods, require qualified staff for sample analysis.

### 2.3. Immunoassay

In order to increase the specificity of antibiotic detection, immunoassays are used relatively often in food laboratories. They are based on the recognition of antibiotics or other compounds by monoclonal antibodies. These assays, however, require labeling of antibodies with molecules that are optically or electrically active. Depending on the kind of label, the assay can be colorimetric [29,30,31], fluorescent, chemiluminescent [32], or electrochemical [33]. Most common in food analysis are immunoassays using enzyme labeling. In this respect, they can be classified on enzyme-linked immunosorbent assay (ELISA), enzyme-linked fluorescent assay (ELFA), fluoroimmunoassay (FIA), and time-resolved fluoroimmunoassay (TRFIA).

ELISA uses an enzyme and chromogenic substrate. In the typical sandwich-based assay, primary capture antibodies are immobilized in microplate ELISA wells. After the addition of the sample containing antibiotics, secondary capture antibodies that are modified by enzymes are added. After the addition of a colorless enzyme substrate, the colored product generated by the enzyme can be detected using spectroscopic methods using a microplate reader [34]. In electrochemical ELISA, the electrically inactive substrate is transformed by an enzyme into a product that is characterized by certain redox properties. This results in the generation of current at a certain potential [33]. ELISA has been adapted for all types of food samples and is often used in the analysis of food contamination by pesticides, other toxins, and antibiotics. Various ELISA kits depending on the detection of the molecules of interest are available on the market. In the case of antibiotic detection, attention should be given to the possible cross-reactivity of antibodies detecting isomers and/or degradation products, leading to the overestimation of the antibiotic concentration in the sample [29]. This problem can be solved by amplifying the signal using secondary antibodies. In this case, ELISA can allow the specific detection of antibiotics with LOD below 1 ng/mL [30]. However, the application of secondary antibodies increases the cost of detection in food analysis.

For routine food analysis, immunochromatographic strip methods are available. For example, PerkinElmer’s AuroFlow™ offers tests for various antibiotic types in raw milk with limits of detection at or below MRL.

### 2.4. Other Analytical Methods for Antibiotic Detection

As we mentioned above, ELISA is the most used technique in routine food analysis. However, other physical methods can be rather useful for antibiotic detection, especially with respect to simplicity of sample preparation. Among them, surface-enhanced Raman spectroscopy (SERS) provides vibrational spectroscopic fingerprints that are related to the chemical composition of the sample [35,36]. However, SERS requires sample pretreatment in order to increase the number of antibiotics relative to other chemical compounds. 

For field applications, microfluidics or paper-based chemical assays can also be used. For example, Sierra et al. [37] reported a microfluidic system that measures chemiluminescent output related to the interaction between aminoglycoside antibiotics and copper ions on a chip. The limit of detection for this method was below 1 ng/mL.

## 3. Nucleic Acid Aptamers

### 3.1. SELEX and the Basic Properties of Nucleic Acid Aptamers

Nucleic acid aptamers (DNA or RNA) are relatively novel structures that, similar to antibodies, have a high affinity toward target molecules, including antibiotics [8]. Aptamers were first independently reported in 1990 by researchers in the USA [38,39,40]. The term aptamer has been introduced by Ellington and Szostak [40] and is a combination of two words—the Latin word “aptus” meaning “to fit” and the Greek word “meros” meaning “the part”. Folded nucleic acids exist in nature (e.g., tRNAs [41]). Their structure allows them to fit into ribosomes carrying specific amino acids for protein synthesis. However, unlike naturally occurring tRNAs, aptamer’s folded conformation allows them to bind to target analytes with specificity and selectivity. In contrast with tRNA, the nucleic acid aptamers discovered in 1990 are synthesized in vitro with combinatorial chemistry. The method of selection of the aptamers—namely, the systematic evolution of ligands by exponential enrichment (SELEX)—was patented by Tuerk and Gold [39]. SELEX starts with a chemically synthesized random library of single-stranded oligonucleotides (composed of 20–80 nucleotides) with large sequence variations (10^13^–10^15^). The oligonucleotides are flanked on both sides by short primers (18–21 bases) that serve as binding sites for polymerase chain reaction (PCR) amplification. In the presence of target molecules (for example, an antibiotic), some of the sequences form complexes with them. Those that do not interact with target molecules are eluted. The complexed oligonucleotides are dissociated and amplified with PCR. This new, much narrower library of oligonucleotides is used in the next cycle of SELEX. After around 10–20 cycles, an aptamer with high specificity to the target can be obtained [42,43]. Once the aptamer sequence is selected, it can be reproduced by chemical synthesis with high accuracy.

Aptamers are also known as chemical antibodies. However, in contrast with antibodies, they are selected in vitro and are more stable. A further advantage of aptamers is the possibility of controlled chemical modification at both DNA ends (5′ or 3′). One end, for example, can serve toward aptamer immobilization on a solid support. In this case, typically biotin, amino, or thiol links are used. This peculiarity also provides oriented immobilization of aptamers, leaving the binding site free for binding with the target. In contrast with aptamers, for oriented immobilization of antibodies, special approaches are required, such as the involvement of A or G proteins, or using only single antibody chains [44]. The other end can be modified by reporter molecules such as fluorescent probes or redox labels that enhance the sensitivity of the detection. Moreover, the chemical modification of aptamers increases their nuclease resistance in complex matrices and, thus, increases sensor stability. Interaction of the analyte with an aptamer is typically based on electrostatic as well as weak van der Walls interactions or the formation of hydrogen bonds. Owing to the nature of these interactions, the aptamer–analyte complex can be dissociated at specific conditions such as increased ionic strength, changes in pH levels, or elevated temperature. This allows sensor regeneration and the ability to reuse it several times.

Another unique advantage of aptamers is the possibility of simple molecular engineering through the exploitation of complementary DNA strands. The supporting part of two different aptamers can be composed of complementary chains that hybridize in a solution, forming aptamer dimers. Two binding sites of the analyte then allow increased sensitivity of detection. This has been demonstrated for the detection of thrombin [45].

Currently, various modifications of the SELEX have been proposed in order to decrease the number of selection cycles required and improve binding affinity. Among them, negative SELEX minimizes unwanted sequences. The counter SELEX or subtractive SELEX can discriminate between closely related target structures. For the selection of small molecules, such as antibiotics, the Capture-SELEX [46] and nanomaterial-based SELEX [47] are also well suited. Detailed reviews of the principles of SELEX and its modifications can be found in [43,48].

An important peculiarity of aptamers is that in aqueous environments, they fold into a 3D structure, thus forming binding sites for specific targets. In contrast to antibodies that usually operate at physiological pH (7.4) and PBS, for aptamers, it is necessary to optimize the buffer properties, including ionic composition and strength. This can affect the binding properties of the aptamers and their dissociation constant, *K_d_*. The lower the *K_d_* value is, the stronger an aptamer–target molecule complex is. The *K_d_* value is typically in the wide range of pM–μM, depending on the target. 

As mentioned previously, the oligonucleotide libraries typically used in SELEX include the 10^13^–10^15^ different sequences composed of 20–80 nucleotides. However, if, for example, a library is composed of oligonucleotides that are 40 bases long, the possible number of unique sequences is 4^40^ = 1.2 × 10^24^. This means that not all possible variations in oligonucleotide sequences are examined when SELEX is performed. Therefore, it is important to examine whether the oligonucleotide sequence selected against a target analyte is the most optimal or whether further increases in its binding affinity, specificity, and selectivity can be achieved through the introduction of post-SELEX modifications, either empirically or with the use of computer-based simulations. 

### 3.2. Computer Modeling of Aptamer–Antibiotic Complexes and Optimization of Aptamer Sequences

As already discussed in previous sections, the identification of aptamers against antibiotics is currently based on the SELEX process or its variants. Nevertheless, and despite the advent of new and improved SELEX protocols, the process remains laborious, with numerous selection rounds required for the identification of new aptamer sequences [49,50].

Irrespective of the SELEX variant employed, the procedure itself has several inherent limitations, which lead to the selection of sequences with suboptimal binding affinity, avidity, specificity, and/or selectivity against a ligand [51,52]. The limited molecular diversities of the initial libraries employed in the SELEX procedure, for example, narrow down the search space for the optimal sequence against a given ligand. In addition, the fact that aptamers tend to acquire stable 2D and 3D conformations can lead to the loss of potentially high-affinity sequences during the PCR amplification step of SELEX [53]. Most importantly, however, the very nature of conventional DNA/RNA libraries (four nucleotides) is a major shortcoming in the evolution of high-affinity aptamers, as traditionally aptamers lack or exhibit weak hydrophobic interactions, limiting their functional contacts and binding properties with a ligand [52].

To address these issues, a number of techniques have been developed that either improve the selection process, modify the nucleic acid chemistry [50,54,55], or enhance an aptamer’s binding affinity and selectivity for a ligand through its post-selection sequence modification [56]. To avoid, for instance, the reduction in PCR efficiency due to the interactions between the nucleotides found in the constant primer and randomized regions or to minimize their contribution to functional motifs, complex primer-free SELEX processes have been developed [57]. Recently, more straightforward methodologies that rely on capillary electrophoresis have been proposed, which can achieve aptamer selection even in a single round [58,59]. Despite their advantages, these novel aptamer generation approaches have not been employed for the selection of sequences against antibiotics, probably since they rely on the use of sophisticated and expensive equipment, thus prohibiting their widespread adoption [60].

As far as the nucleotides themselves are concerned, biased or patterned initial libraries have been proposed, where, for example, a slight shift to G and C led to the formation of structures with more stems and more complex 3D structures, compared with an A and T shift [61,62,63,64]. To expand the genetic code beyond the four natural nitrogenous bases, modified or artificial nucleobases have been introduced that allow a much more complex repertoire of interactions to be achieved between the aptamers and its ligand [65,66] and also confer resistance to nuclease degradation [67]. Neither of these optimization techniques has extensively been employed in the case of aptamers for the detection of antibiotics, with only a few reports on the utilization of modified nucleobases to resist endonuclease degradation. De-los-Santos-Alvarez et al. showed that a 2′-O-methyl derivative of the RNA aptamer against neomycin B resisted endonuclease degradation and also maintained its affinity against this antibiotic [68]. Interestingly, in another publication on the development of an aptasensor for tobramycin in human serum, a comparison of a partially and fully O-methylated modified RNA aptamer revealed that the fully O-methylated aptamer had a lower dissociation constant than the partially methylated one [69]. Similarly, the use of 2′-fluoro-2′-deoxyribonucleotide modified pyrimidine nucleotides in an RNA aptamer against danofloxacin allowed the discrimination of this antibiotic from other fluoroquinolones [70].

Further enhancement of an aptamer’s binding affinity, selectivity, and/or specificity can be achieved through its post-selection sequence manipulation [51]. Toward this goal, a number of techniques have been employed, including the random or site-directed introduction of mutations, truncations, and even joining of different or identical aptamers to achieve bi- or multivalence and increase the interactions with a ligand [52,71,72,73]. With regard to aptamers targeting antibiotics, numerous examples of such post-SELEX optimization studies have been published. Kwon et al. truncated a 76 mer ssDNA against oxytetracycline to an unusually short 8 mer ssDNA that has been employed for the detection of oxytetracycline, both colorimetrically [74] as well as with a SERS platform [75]. Furthermore, successive truncations of a 79 nt long aptamer selected against tobramycin resulted in a 15 nt sequence that exhibited higher affinity than the original sequence [76]. Similar improvements to the dissociation constant in the aptamer selected by Soheili et al. against streptomycin were achieved through its truncation [77]. More recently, four candidate sequences that could bind to kanamycin were selected for the fabrication of a structure-switching biosensor via restriction-enzyme-based SELEX, which were also further truncated to increase selectivity over other aminoglycoside antibiotics [78]. 

Collectively, the techniques discussed above have immensely contributed to the advancement of the field of aptamer research and applications. However, in comparison with antibodies, aptamer technology still appears to be in its infancy, as the current overall success rate of SELEX is far from satisfactory [52]. The identification of new sequences, and, most importantly, their engineering to tailor their affinity, specificity, and selectivity, remain inefficient, as they largely depend on a trial-and-error screening of a great plethora of possible modifications and SELEX technologies. Computer-based aptamer selection and rational design, on the other hand, could potentially revolutionize research on functional nucleic acids by allowing aptamers to attain their full potential.

**Figure 1:**
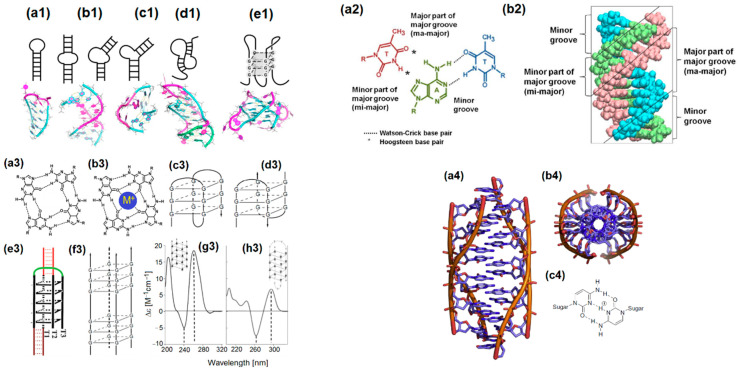
**Upper left:** Secondary structural elements and motifs found in nucleic acids including aptamers: (**a1**) stem-loop; (**b1**) internal loops; (**c1**) bulge; (**d1**) pseudo-knot; (**e1**) G-quadruplex [87]. **Upper right**: Triple-helix structures: (**a2**) the bases of the triplet of T–A*T are indicated by light blue, light green, and pink; (**b2**) the structure of Ts1 is depicted by the van der Waals model. First (5′-TTTTTTTCTTCT-3′), second (5′-AGAAGAAAAAAA-3′), and third (5′-TCTTCTTTTTTT-3′) strands are indicated by light blue, light green, and pink, respectively [88]. **Lower left:** The structure of (**a3**) G-quartet is formed by four guanines; (**b3**) the G-quartet is stabilized by chelating a cation (such as Na^+^, K^+^, or TI^+^). G4 structures are assembled with (**c3**) one, (**d3**) two, (**e3**) three, or (**f3**) four strands of DNA. Representative CD spectra of (**g3**) a parallel G4 and (**h3**) an antiparallel G4 structure. Reproduced with permission of Elsevier from ref. [89]. **Lower right:** C rich sequences can form i-motifs under acidic conditions: (**a4**) structure of the d(TC5) intermolecular i-motif (PDB ID: 225D); (**b4**) top view of d(TC5); (**c4**) a hemi-protonated cytosine–cytosine^+^ base pair. Reproduced with permission of Elsevier from ref. [90].

Initial attempts to optimize the selection process with the use of computer-based simulations focused on the identification of common motifs in the “tertiary” structure of published aptamers, in order to pattern or bias SELEX libraries to improve the selection efficacy [79,80]. In silico prediction of an aptamer’s structure is based on calculating minimal free energy and/or portioning functions and using 2D structural elements data as input for deciphering an aptamer’s 3D folding. Many software packages (e.g., MEME Suite, MEMERIS, GLAM2, mFold, Aptamotif, ViennaRNA, RNAStructure, RNAsoft, CONTRAfold, NUPACK, Eternafold, etc.) have been developed to rank, cluster, and identify common motifs in the vast number of sequences that result from high-throughput SELEX processes [81,82,83,84,85]. These motifs include pseudoknots, G-quadruplexes, and, most commonly, stem–loop structures such as hairpin loops, bulge loops, and interior loops that can form even more complex structures such as kissing hairpins (Figure 1) [86,87,88,89,90].

Modeling must consider the flexibility of the phosphodiester backbone and all possible base pairings, including noncanonical base pairing, as well as the influence of hydrophobic interactions [91]. Tertiary structure prediction is even more complex and is based on sequence analysis or homologous sequence structures from databases. Despite the fact that both approaches have limitations, considerable progress has been made recently, especially in predicting RNA structures with software programs such as RNAComposer, BARNACLE, FARNA, and MC-Fold|MC-Sym Pipeline, followed by their conversion to DNA using 3DNA or VMD [92,93,94]. Some of these programs can be directly applied for the in silico maturation of an aptamer through mutagenesis and truncation. As an example, COMPAS can find motif combinations that are essential for binding and non-conserved sequence positions that are less critical, which could be removed [95]. AptaMut, on the other hand, predicts the affinity achieved by mutated sequences [96].

Once an aptamer’s 3D structure has been predicted, docking simulations can be undertaken that are able to reveal the steric and energetic properties of each aptamer–ligand complex, which, in turn, are based on several non-covalent interactions, including ionic interactions, hydrogen bonds, van der Waal’s forces, hydrophobic interactions, and base stacking interactions [97,98]. These forces, in conjunction with shape complementarity and internal DNA energy, play important roles in establishing the way an aptamer interacts with its ligand [99]. It is worth stressing that the quality of docking simulations with the use of software packages such as DOCK6.9, AutoDock Vina, ZDOCK, HADDOCK, or rDock depends on the accuracy of 2D and 3D aptamer structure prediction [100,101,102]. Regrettably, structural studies for aptamer–ligand interactions are still the bottleneck in this field, as both X-ray crystallography and NMR are challenging techniques to employ since the experimental determination of 3D DNA or RNA structures is notoriously difficult [103,104,105]. Inaccuracies due to the lack of structural data on aptamers and the conformations they adopt when complexed with their ligands are further aggravated by the fact that most of the docking algorithms have been initially developed for proteins and protein–protein interactions [106]. This results in the introduction of progressively larger, compounding errors in subsequent pipelines. On the bright side, these algorithms are constantly being refined through the release of new and improved software [107,108,109]. Finally, special emphasis should be placed on deep learning methods, whose efficiency has outperformed other methodologies in 3D RNA structure ranking [110,111] and have begun to appear in the computational aptamer field as well [112,113].

**Figure 2:**
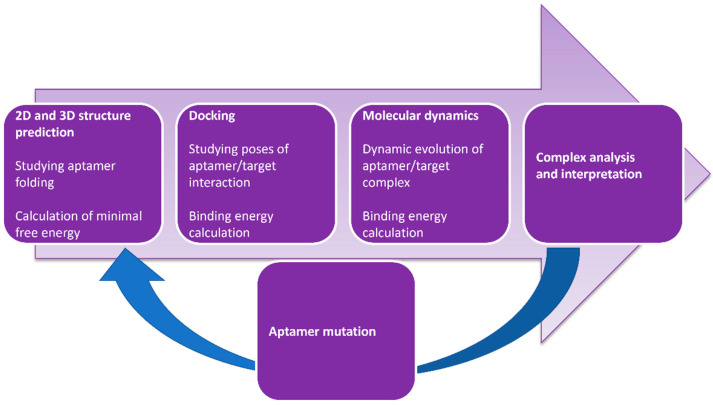
Schematic overview of a simulation-based workflow for the prediction of an aptamer’s structure and its interactions with its ligand. Through rational introduction of mutations or the use of genetic algorithms, the sequence of an aptamer can be altered, and a new round of simulation-based prediction of its improved affinity for its ligand can be undertaken [92].

Although docking simulations are powerful techniques to elucidate 3D structures that are stable with respect to energetics, ideally one would also study the dynamics of these structures over time. MD simulations generate a conformational ensemble of nucleic acid structures at equilibrium and provide detailed atomic motions that aid in understanding structure–function relationships in an aptamer [112,114]. Due to the advantages of incorporating MD simulations into a computational pipeline, an increasing number of published studies report the use of software packages such as NAMD, AMBER, and GROMACS [101,115,116]. Ultimately, computational methods could be applied to predict the interactions between an aptamer and its ligand and, hence, refine the nucleic acid sequence to improve the binding affinity and specificity, in addition to being applied for the de novo selection of aptamer in silico [114,116]. An overview of the workflow followed for the simulation-based prediction of an aptamer’s interactions with, and affinity for, its ligand is shown in Figure 2.

The use of computer-based simulations is increasingly gaining interest in the field of aptamer research against antibiotics [99]. Ilgu et al., for example, have combined computational and experimental approaches to understand if aptamers with different sequences targeting aminoglycoside antibiotics share structural and dynamical features. Interestingly, they concluded that the ligand drives the structure of an aptamer [117]. Refinement of the native aptamer against sulfadimethoxine was attempted with the use of MD simulations by Khoshbin et al. [118]. The optimized sequence exhibited higher binding affinity but also enhanced selectivity against sulfadimethoxine. Domin et al., on the other hand, used secondary RNA structure prediction software RNAFold to design RNA riboswitches against tetracycline and streptomycin [119]. Finally, in an attempt toward the in silico selection of aptamers against gentamicin, neomycin, and tobramycin, Chushak and Stone proposed a two-step computational approach that decreased the RNA sequence search space in a selection pool by up to five orders of magnitude [120]. De novo selection of aptamers based on computer simulations was demonstrated by Khavani et al., who selected two aptamers against neomycin B that displayed high affinity for the latter and also theoretically demonstrated the type of interactions and forces that evolve in the aptamer–ligand complex [121]. The published studies previously discussed illustrate the power of computer-based simulations in the field of aptamer research, which is set to revolutionize aptamer selection and refinement against antibiotics.

## 4. Principles of Biosensors and Electrochemical Methods of Antibiotic Detection

Biosensors are portable analytical devices that utilize biochemical recognition events converted into the physical–chemical signal detected by appropriate transducers [122]. As we already briefly mentioned in the Introduction section, biosensors are mostly classified in accordance with the biorecognition element and transduction principle. Thus, enzyme [123,124], immuno- [125,126], and DNA sensors [127,128] are used with appropriate biorecognition elements attached to the transducer interface or placed near them. Aptasensors containing aptamers specific to a certain analyte belong to the DNA sensors family [129].

Interaction of an aptamer with its target is ascribed as a reversible reaction so that the maximal signal of the biosensor corresponds to reaching equilibrium of the aptamer–analyte binding. In this sense, aptasensors are methodologically similar to immunosensors, in which the reaction of antigen and antibody is detected. In both kinds of biosensors, no changes in the chemical structure of an analyte are expected, and the recognition event is reversible from the point of view of classical physical chemistry. Although the equilibrium mentioned is shifted toward the complex analyte–aptamer, in some cases, it can be returned to non-bonded reactants by the addition of electrolytes or gentle heating of the solution. 

Proceeding with the comparison of aptasensors with immunosensors, it should be noted that the analysis of small analyte molecules such as antibiotics has limitations similar to the ones observed in the case of hapten immunosensing. Sandwich assays with the use of two antibodies, popular in conventional immunochemical assays, are not applicable in the case of aptasensors devoted to antibiotic determination. The latter types are just too small and do not offer an appropriate epitope for the capturing and signaling of antibodies/aptamers [130]. Meanwhile, it is possible to create sandwich-like reaction schemes with labeling DNA partially complementary to an aptamer that is binding to a terminal fragment of the aptamer–analyte complex and, hence, attaching a label to the electrode interface [131].

Other reaction schemes have been proposed for small analytes, from which indirect competitive assay, described in the following sections, is the most popular [132]. Aptasensors offer more opportunities for biochemical amplification of the signal based on specific mechanisms of DNA recycling, DNA walking machine, etc. Being extremely sensitive, such protocols require deep modification of the DNA strands and application of many auxiliary agents such as DNA hairpins, specific enzymes (exo- and endonucleases), etc.

Strictly speaking, indirect competitive assays and biochemical amplification schemes mentioned above do not fully comply with the definition of a “biosensor” because target interactions occur in solution in homogeneous conditions and not on the transducer interface. Moreover, total measurement time, including that of the incubation and washing steps, can take several hours. This does not meet the requirements of detection in real time, even if a miniature platform is used. Nevertheless, such biosensing devices are included in consideration as a proof of concept of technological solutions and methodologies that are applicable in the future for the elaboration of point-of-care devices [133] or microfluidic chips [134].

### 4.1. Aptamer Imobilization Protocols

Aptamers are either covalently bonded or physically adsorbed on the electrode surface, together with some auxiliary components, such as mercaptohexanol that prevent the adsorption of the analyte to the naked gold surface. In addition to conventional immobilization protocols, based on which aptamers are directly deposited onto the sensor surface, hybridization of aptamers with already immobilized complementary DNA strands (so-called capture DNAs) has also been reported [135]. In some cases, such an immobilization strategy offers more opportunities for the generation of the signal toward analyte–aptamer binding. Appropriate examples are given below. Covalent binding of the aptamer is mostly achieved via terminal functional groups and is closely related to the nature of the underlying material. Thus, thiolated aptamers are immobilized on golden particles or bare Au electrodes via spontaneous formation of Au–S bonds [136,137], and aminated aptamers are covalently bonded to the carboxylated supports such as carbon nanomaterials [138]. Recently, electron grafting of the electrode with diazonium salt formed from p-aminophenylacetic acid or p-aminobenzoic acid has become popular [139]. In this method, the precursor compound is first treated with nitrite to form a diazonium salt, which is then electrodeposited on the electrode. Finally, carboxylic groups are involved in the reaction with carbodiimide to immobilize aminated aptamers.

**Figure 3:**
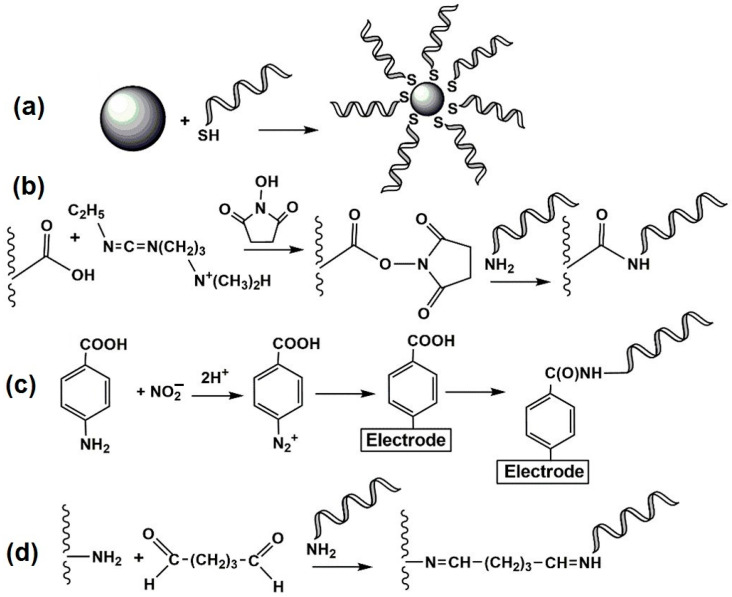
Aptamer immobilization protocols applied in the electrochemical aptasensors: (**a**) interaction of thiolated aptamer with Au nanoparticles/bare Au electrode; (**b**) carbodiimide binding to carboxylated support; (**c**) electrografting with diazonium salt generated from aromatic amino group; (**d**) cross-binding with glutaraldehyde of aminated aptamer and carrier.

Cross-linking with glutaraldehyde is used for the same purpose [140]. Glutaraldehyde binding is advantageous, for example, in the case of application of aminated carriers, e.g., poly(amidoamine) (PAMAM) dendrimers that allow an increase in surface density of the aptamers. Principal schemes of the immobilization protocol are outlined in Figure 3.

Covalent immobilization schemes are usually used for the aptamer-based detection of rather large analyte molecules. In the case of antibiotics, the steric hindrance of the reaction should not significantly affect the performance of the aptasensor. However, many biochemical amplification steps require the interaction of complementary parts of the DNA strands and of the aptamer; hence, they also require accessibility and flexibility of rather long DNA fragments in the aptamer strand [141]. In such aptasensors, hairpin DNA strands or aptamers are attached to the transducer, and interactions with an analyte or auxiliary DNA result in a conformational shift to linear configuration. In many cases, the problems related to steric limitations of interactions on the electrode interface can be minimized by the introduction of linear alkyl or oxyalkylene (oxyethylene) tags that shift the recognition rection from the electrode surface. They are rather easily introduced in the aptamer structure. Many of such derivatives are commercially available.

Whichever method of site-specific immobilization is followed, non-specific adsorption is prevented by covering the bare support/electrode surface with a monolayer of hydrophilic species such as 1-mercaptohexanol in the case of thiolated aptamers. Such treatment makes aptamers acquire an orthogonal position against the support plane and promotes interaction with bulky analytes or auxiliary DNA sequences.

### 4.2. Electrochemical Signal Measurement Modes

Any change in the physical-chemical parameters related to the analyte binding can be exploited toward its quantification. Among all the protocols described (e.g., mass sensitive [142,143], colorimetric [144], fluorescence biosensors [145], etc.), electrochemical methods are most frequently applied for this purpose. This is due to the advantages offered by electrochemical sensors, such as (1) rather high sensitivity and selectivity of signal measurements within a reasonable time interval; (2) a well-elaborated theory of electrochemical sensors, intuitively understandable interpretation of the signals achieved; (3) low requirements in terms of measurement conditions and sample pretreatment, and a limited number of potential interferences in biological samples; (4) the simple design of measurement equipment, which is easily adapted to a portable format and application outside the laboratory; (5) low cost of the auxiliary components used in electrochemical sensors’ assembly and measurement.

**Figure 4:**
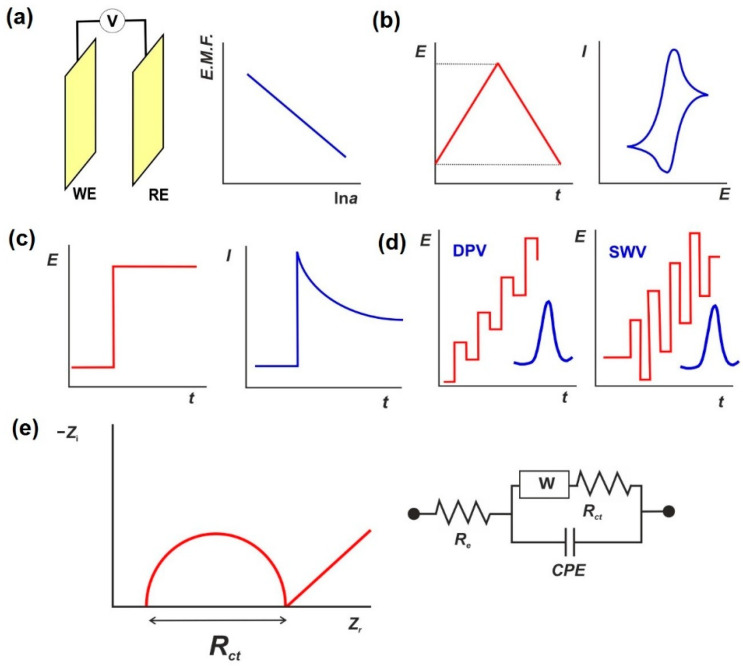
Electrochemical sensors used as transducers of aptasensors: (**a**) potentiometric cell: WE—working electrode, RE—reference electrode, V—voltmeter; (**b**) cyclic voltammetry; (**c**) amperometry; (**d**) differential pulse (DPV) and square wave (SWV) voltammetry. Red line illustrates the shape of current peak obtained; (**e**) electrochemical impedance spectroscopy; the Nyquist diagram and equivalent circuit correspond to the double-electric layer. *R_e_* is electrolyte resistance, *R_ct_* is charge transfer resistance, *W* is Warburg impedance, and *CPE* are constant phase element.

Although the principles of electrochemical measurements are well known, in this section, we shortly discuss them in relation to aptasensors for antibiotic detection. Appropriate schematic outlines of signal generation are presented in Figure 4. 

**Potentiometric sensors** utilize ion-selective electrodes, whose potential is sensitive to a certain ion present in the solution. Electromotive force (e.m.f.) is measured with a reference electrode, whose potential is independent of the analyte concentration. In ideal conditions, the signal of an ion-selective electrode is proportional to the logarithm of the activity of the potential determining ion. Measurements with potentiometric biosensors are performed in open-circuit mode, with no external source of electric power. Meanwhile, the slope of the calibration curve described by Nernst’s equation inversely depends on the charge of the analyte. Aptamers are polyanionic substances because of the presence of phosphate residues in the backbone. Their interaction with small molecules cannot seriously affect the potential of the aptasensor. Nevertheless, some examples of potentiometric aptasensors have been described [146,147,148]. Among them, carbon nanomaterials are mostly used, and changes in the surface charge are monitored in the presence of polycations being released from the surface upon the aptamer–analyte interactions.

Constant current voltammetry, on other hand, exploits the polarization of the working electrode used as an aptasensor transducer. The current produced at the working electrode is attributed to the redox reaction of a label or diffusional free redox probe. Under certain conditions, the relationship between the current and analyte concentration is linear. Thus, voltammetry offers higher sensitivity and accuracy over potentiometric sensors. In its simplest form, the potential is maintained constant and the current–time dependence is recorded (in chronoamperometry or amperometry mode). Amperometric aptasensors are relatively simple and are mostly considered for application outside chemical laboratories (point-on-demand or point-of-care biosensors).

In **linear sweep voltammetry** (LSV), the applied potential is controlled against a reference electrode and is linearly increased in time. In **cyclic voltammetry** (CV), triangular scanning of the potential is applied so that the potential is first increasing to its return value and then decreasing back to the initial potential with the same scan rate. CV is one of the principal tools used for the exploration of reactions occurring on an electrode. Variations in the potential scan rates and pH levels make it possible to determine the stoichiometry of the electrode reaction (number of electrons and hydrogen ions transferred), its reversibility, a possible influence of electrocatalysts, etc. The shapes of the peaks on voltammograms corresponding to certain species reflect the relative contribution of the electron and mass transfer phenomena expressed in terms of electrochemical reversibility of the electrode reaction. The assembly of an aptasensor is usually examined with the use of cyclic voltammetry in which changes in the voltammetric signals of the label/probe are considered lines of evidence of the successful stepwise modification of the surface of an aptasensors. Thus, deposition of electrochemically inactive modifiers, such as functionalization chemistries, results in a decrease in both the peak current and the reversibility of the electron exchange, which is usually examined with the use of either ferri–ferrocyanide or methylene blue as redox probes in solution. Their reversible behavior over a wide range of pH values is stable toward chemical oxidation with dissolved oxygen. Both can also be introduced in aptamers and auxiliary DNA strands with the same methods previously described for aptamer immobilization.

While LSV is used for the electrochemical characterization of an aptasensor’s assembly, aptamer–analyte interactions are usually examined with a pulsed technique. **Differential-pulse voltammetry** (DPV) and **square-wave voltammetry** (SWV) both use a combination of linear scans of constant current potential and small pulses that shift the system from equilibrium [149]. The idea is that the relaxation of faradaic currents is slower than that of capacitive currents so that their ratio becomes much higher at the end of the pulse than in the LSV experiments. This results in an increase in the sensitivity of the method and a decrease in the concentrations determined by about 1–2 orders of magnitude. The signal recorded with pulsed techniques is similar to the derivate of the conventional voltammetric *I*–*E* curve and has a symmetrical bell shape. Higher sensitivity, however, comes at the cost of lower descriptiveness of the mechanism of electrode reaction.

**Electrochemical impedance spectroscopy** (EIS) assumes polarization of the electrode at a constant potential corresponding to the equilibrium of the electron exchange [150,151]. A small sinusoidal alteration of equilibrium by the application of small potential shifts is conducted with the frequencies varied from 0.01 Hz to 100 kHz, and the impedance (ratio of alternating potential to alternating current) is measured. Interpretation of the dependencies is based on the assignments of electric chain components to the double electric layer on the electrode interface. Results are usually presented as Nyquist plots using imaginary and real parts of impedance. The diameter of the semicircle at high frequencies is assigned to the charge transfer resistance. In aptasensors, an equimolar mixture [Fe(CN)_6_]^3−/4−^ is used in EIS experiments (so-called faradaic EIS), although intrinsic redox activity of modifiers can also be applied. The measured charge transfer resistance depends on both the electrostatic repulsion of the redox probe and the lowering of its diffusion rate due to the analyte binding. The small size of antibiotics is compensated for by the aptamer’s structural rearrangements upon recognition of an analyte. The formation of so-called G4 quadruplexes or the transfer from hairpin to linear configuration sufficiently affects the EIS parameters to allow the detection of sub nanomolar concentrations of the analytes.

**Chemoresistors** utilize the dependence of the resistance of some materials on the chemical content of the sample tested. Appropriate assemblies utilize graphene or some polymers deposited between two metal electrodes, as in the case of interdigitated electrodes manufactured on plastic or glass support [152]. Alternatively, chemoresistor principles can be utilized with field-effect transistors, where single-walled carbon nanotubes are placed in the gap between the drain and the source areas of the transistor [153].

### 4.3. Aptasensor Signal Measurements 

The difficulty associated with the detection of electrochemically inactive compounds is one of the important drawbacks of electrochemical sensors, including electrochemical biosensors. Aptamers themselves do not exert redox activity. Although direct oxidation of guanine and adenine residues has been described for native DNA [154], it is not used for aptasensor signal detection due to the rather strict measurement conditions required and the low sensitivity of the response due to the rather short nucleotide sequences in the aptamer molecules. Instead, three protocols have been elaborated and successfully used: (1) determination of the redox signals from labels with which the aptamer are modified and that exert high redox activity; (2) measurements of the electrochemical signals of diffusionally free, small molecules capable of electron transfer (redox probes or redox indicators); (3) measurements of the changes in the redox properties of auxiliary agents placed on the electrode prior to or together with an aptamer. 

Each protocol has its own drawbacks and advantages. Thus, the use of labels offers a reliable signal because the concentration of the label is directly related to the number of the aptamer molecules modified with the label. In this case, changes in the label signal caused by analyte binding can be attributed to the steric factors affecting the contact of the label with an electrode and, hence, the electron transfer. Aptamer–analyte interactions can affect the configuration of the aptamer and the accessibility of the label for the electrode reaction. In the case of bulky analytes, their introduction in the surface layer containing aptamer molecules will also diminish the signal from redox molecules arriving at the electrode surface. As we already mentioned above, methylene blue [155,156] and ferrocene [157,158,159] are mostly used as labels in aptasensors for small molecules detection. However, the chemical modification of the aptamer increases the cost of the aptasensor. 

Diffusionally free redox probes change their surface concentration and/or reactivity due to the capturing of electrochemically inactive analytes and/or electrostatic interactions within the aptamer layer. In the first case, specific binding decreases the rate of diffusion of the redox probe to the electrode. In the second case, electrostatic repulsion–attractions between the redox probe and the sensing surface occur. Thus, the most popular redox probe, ferricyanide ion, interacts with negatively charged phosphate residues of the aptamer backbone. Both mechanisms can affect the signal simultaneously, and their relative contribution depends on the aptasensor assembly, analyte structure, and signal measurement conditions. In the case of small analyte molecules, diffusional limitations seem less important. However, in many cases, aptamers are folded in the presence of analytes. Their density is increasing with the analyte concentration; hence, the current of redox probes inversely depends on the quantities of the target species. In addition to the ferricyanide ions, methylene blue [160,161] and thionine [162] are often used as redox probes due to their ability to interact with double-stranded and single-stranded DNA molecules, in addition to showing remarkable voltammetric signals. In many protocols, hybridized double-stranded structures are either formed or dissociate and the signals of the above-mentioned redox probes changed with such events. It is important that the number of probe molecules released/bonded in the recognition event exceeds that of the label so that sensitivity of the signal is higher. It should be also noted that the same probes can be accumulated on the surface of some carbon nanomaterials or metal–organic frameworks (MOFs) which serve as supramolecules labels with multiplied sensitivity of the signal toward certain analytes.

In some cases, the aptamer–analyte reaction affects the intrinsic redox activity of modifiers deposited on the electrode prior to, or together with, the aptamer. In the case of redox-active polymers, the signal is related to the influence of aptamer–analyte interaction on the charge separation and hence on the equilibrium of electron exchange in the layer [163]. Most of the electropolymerized materials are positively charged in an oxidized state and neutral in reduced form. Thus, the increased negative charge of the phosphate groups of the aptamer sequence or its shielding due to folding or analyte binding affects the potential of the electropolymerized layer or generates current from the transfer of reduced/oxidized forms of the polymer.

## 5. Electrochemical Aptasensors for Antibiotic Detection

Table 2 summarizes the published articles on electrochemical aptasensors for antibiotic determination in the period 2018–2022. Earlier periods are covered in a number of excellent reviews devoted to individual antibiotics (aminoglycosides [76,164], chloramphenicol [165,166,167], tetracyclines [168,169], and kanamycin [170]) or to the application of certain components (Au particles [171] and nanomaterials [172,173,174]). For each antibiotic, the references are placed in chronological order. The units used for the limit of detection and the dynamic range achieved with each aptasensor are the ones stated in the original publications. It should be noted that, in some cases, the sequences reported in Table 2 include spacers and additional recognition sites required for certain signal generation protocols, and that is why the aptamer sequence is underlined.

All of the reported aptasensors can be further subdivided into two groups depending on the changes in the signal upon analyte recognition. In signal-on aptasensors, the signal increases with increasing analyte quantities. In signal-off aptasensors, the signal is decreasing from an initial baseline value with increasing analyte content. Signal-on aptasensors are preferable because the accuracy of the detection of small shifts in the signal in the area of low analyte concentrations is often higher than the decrease in maximal signals in the case of signal-off aptasensors. It should also be noted that the decrease in the signal recorded with signal-off aptasensors depends on the resolution of the measurement. 

### 5.1. Surface-Layer Permeability Assessment 

The formation of an aptamer–analyte complex provides sensitive determination of antibiotics only in the case of significant structural rearrangements of the aptamers and the formation of a dense layer hindering the access of redox probes to the electrode interface. In conventional schemes, the rate of electron transfer becomes lower and the signal decreases with increased analyte concentration (signal-off aptasensor). The signal can be recorded either with EIS or DPV/SWV, the results of which should coincide. Precision and the background signal resolution of the measurement are highly dependent on the regularity of the surface layer and the density of immobilized aptamers.

In most cases, the LODs of biosensors indicated in Table 2 are below MRL. For example, the MRL of kanamycin in milk is 0.4 μg/kg (Table 1), which corresponds to 0.8 nM or 0.414 ng/mL (considering the milk density of approximately 1035 kg/m^3^ at 20 °C). MRL for tetracycline is 100 μg/kg, which corresponds to 225 nM or 103.5 ng/mL and is substantially below LOD (Table 2).

**Figure 5:**
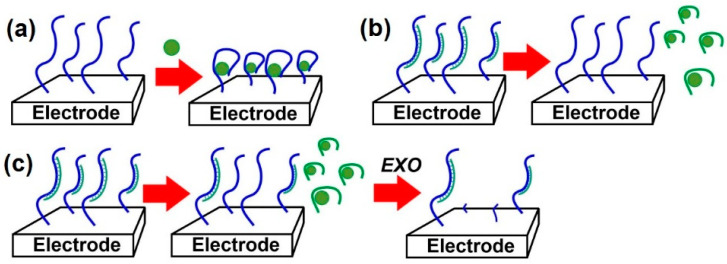
Electrochemical aptasensors for antibiotics based on the monitoring of the surface layer permeability: (**a**) aptamer folding after analyte recognition; (**b**) analyte removes aptamer from its hybrid with capturing DNA attached to the electrode; (**c**) Remained single-stranded DNAs are additionally cleaved with exonuclease.

To further improve LOD, the aptasensors based on Au nanoparticles obtained via electrodeposition on bare electrodes or carbon nanomaterials have been extensively employed [171]. Furthermore, the use of auxiliary DNA strands complementary to the aptamer can be immobilized onto the electrode and used for aptamer capturing through its hybridization with them. Recognition of an analyte by the aptamer results in signal increases, as the aptamer is released from the surface so that the density of the surfaced layer becomes lower (similar to the more conventional strand displacement protocols described below). Additional amplification has been demonstrated by treating the capturing DNA remained after the aptamer release with Recjf exonuclease that cleaves the DNA sequence and results in further decreases in the resistance of the charge transfer [264]. The opposite effect can be achieved by the reaction of auxiliary DNA with protein specifically bonded to single-stranded DNAs [245,263]. The indirect competitive assay is realized in [246]. In this study, the aptamer is first mixed with the analyte, and then the mixture is added to the electrode covered with a monolayer of auxiliary DNA complementary to free aptamer molecules. The hybridization product is then cleaved with an exonuclease, and changes in the permeability of the layer are controlled using EIS with [Fe(CN)_6_]^3−/4−^. Schemes of the aptamer-based monitoring of the permeability of the surface layer are presented in Figure 5.

### 5.2. Lalbeled Aptamers/DNA Strands

**Figure 6:**
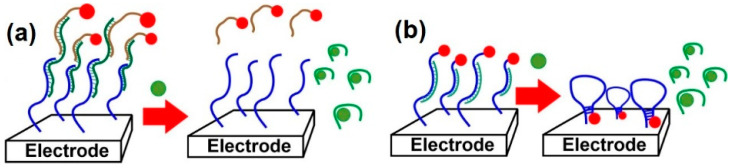
Label-based detection of antibiotics by electrochemical aptasensors: (**a**) aptamer is partially complementary to capturing DNA attached to the electrode and signaling DNA-bearing label. Interaction with analyte releases aptamer–analyte complexes, and this results in removal of labeled DNA from the electrode interface; (**b**) aptamer forms duplex with partially complementary DNA attached to the electrode and modified with label at its opposite end. Interaction with analyte removes aptamer from the duplex and allows DNA to convert to hairpin configuration with label near the electrode surface.

As already mentioned, the small size of antibiotics does not allow the utilization of conventional sandwich assays with the formation of triple complexes consisting of aptamers and capturing and signaling DNA strands attached to the electrode. Nevertheless, a reverse scheme is quite successful (Figure 6), according to which an aptamer is hybridized with short complementary DNA strands, while interaction with an analyte destroys the complex and results in the release of the labeled DNA from the electrode. As a result, the label signal decreases with the increasing concentration of the analyte. Alternatively, signal-on aptasensors can be designed using a DNA–aptamer complex, in which the auxiliary DNA bears a terminal label and can self-hybridize, forming a hairpin configuration. Upon analyte binding, the aptamer is removed from the complex, and the auxiliary DNA changes its configuration. Thus, the terminal label is in closer proximity to the electrode surface, and therefore, the signal increases.

Additional amplification of the signal can be achieved by using nanomaterials. Thus, metal–organic frameworks (MOFs) can act as nanocontainers through their saturation with redox probes exerting their own signal, which is multiple times higher than that of a single molecule [176,180,188]. Alternatively, quantum dots (QDs) can be digested after attaching to the aptamer–analyte complexes, followed by the determination of the products (Cd^2+^, Pb^2+^ ions) with conventional stripping voltammetry [176,181,182,187]. It should be noted that the use of such nanocontainers is relatively limited due to the uncertainty introduced in the quantities of the redox-active species released per analyte binding, which results in lower accuracy of the measurement results.

### 5.3. Biochemical Amplification Protocols

Biochemical amplification is based on the use of biocatalysts (enzymes and DNAzymes) that amplify the signal due to catalytic recovery of compounds involved in the signal generation. The simplest protocol of such amplification is based on the ELISA platform where an enzyme (mostly horseradish peroxidase) attached to the signaling DNA strand, either directly or via avidin–biotin binding is employed. HRP activity is measured using the hydroquinone–benzoquinone system and H_2_O_2_ by the current of the hydrogen peroxide reduction–oxidation [183,199,214,248]. In more sophisticated protocols, amplification cycles have been used with several auxiliary DNA strands in hairpin configurations that are involved in the strand displacement with the recovery of analyte–aptamer interactions in the presence of trigger DNAs interacting with aptamer [175,269]. Similar to these, a hybridization chain reaction is activated that results in the formation of long ds-DNAs on the electrode surface, followed by the accumulation of methylene blue as an indicator of hybridization events [178]. The description of such mechanisms of signal amplification is given in Table 2. Its detailed consideration is out of the scope of this review but can be found in another review [270]. The use of biochemical amplification allows ultralow concentrations of analytes to be detected that are significantly lower than their limited threshold levels in food and feed. Considering the strict requirements of the measurement requirements and the necessity of heating/washing steps, such aptasensors are mostly considered as a proof of concept and have limited prospects in real-sample assays. Indeed, determination of the femtomolar concentration of antibiotics within several hours is hardly something that is required. The assessment of the metrological characteristics of aptasensors utilizing biochemical amplification is complicated due to the existence of two separate steps of analysis. The detection of redox probes on the electrode is rather conventional and easy for calculation of relative deviation or confidence intervals. However, homogeneous steps that precede instrumentation use are not assessed from the points of view, repeatability, or uncertainty of the results. Probably, for this reason, the announced metrological characteristics of such aptasensors seem overly optimistic. At least, careful consideration of the influence of all the steps of analysis is required.

### 5.4. Real-Sample Analysis

Although antibiotics are mostly intended for the medical treatment of various diseases related to pathogenic bacteria, appropriate aptasensors are used and developed for testing veterinary products or food quality. The increased interest in milk analysis (about 80% of all the cases of real-sample testing), for example, is related to the high level of application of veterinary drugs in cattle farms. Overdoses and accumulation of antibiotics in meat and milk are dangerous from the point of view of allergies and the immune status of healthy people. Meanwhile, the control of pharmacokinetics (residual quantities of the drugs in urine) is still out of reach for researchers. To some extent, this may be due to the strict requirements of medical investigations related to bioethics. Determination of antibiotics in fresh and tap waters seems interesting from the point of view of future contamination of the waters with medicines. However, the present level of drug residues found in basins remains rather low to affect human health. Moreover, the matrix effects of such samples are negligible, and the calibration plots are commonly very similar to those obtained with standard solutions. Regarding interferences present in milk and meat, most of the studies apply protocols of sample treatment derived from those used in chromatographic analysis. They include sedimentation of proteins and other biopolymers, filtering, and dilution of the samples. Few of the articles cited provided an independent analysis of data obtained with reference methods. Most of the aptasensors described do not examine surface regeneration and reusability. This might be related to the difficulties in the dissociation of the aptamer–analyte complexes formed on the transducer interface and changes in the affinity of target interaction after recovery. Such an approach is rather reasonable for medical diagnostics, where possible biological contamination of the biosensor and increased probability of false results are considered as arguments in favor of single uses of biosensors. However, in the case of antibiotics detection in milk, water from various sources, and meat, the above-mentioned arguments are less significant, and sensor reusability should be aimed for this purpose.

### 5.5. Recent Trends in Aptasensors Design

Although most of the details concerning the design and performance of electrochemical aptasensors are presented in Table 2, it seems important to highlight the most urgent trends in their development and application. Among modifiers intended for aptamer immobilization and signal generation, MOF-/COF-based architectures are intensively studied [176,180,188,189,218,221,235,265,266,270]. They are rather easily synthesized by hydrothermal methods and can either accumulate reactants including aptamers or serve as carriers for labels. Due to their relatively large size and high amount of metal ions, potentially capable of redox conversion, such MOF labels can significantly amplify the signal. Releasing metals from the MOFs after their separation in the aptamer–drug complex is a universal way for sensitive and specific detection of target species, irrespective of their electrochemical activity and size. MOFs offer a great variety of properties, depending on the organic microenvironment of metal atoms and synthesis protocols, but only well-known representatives such as UiO-66–NH_2_ [176] have been applied to date. Thus, further progress in this direction can involve the enhancement of MOF structures applied in electrochemical aptasensors. To some extent, this can be also referred to QDs. They are both used in optical detection systems, ECL biosensors, and electrochemical aptasensors. In the latter case, QDs mostly consist of carbon nanomaterials: QDs do not worsen the electroconductivity of the surface layer but improve the surface density of the aptamers on the electrode interface and, hence, increase the signal-to-noise ratio by partial suppression of undesired adsorption of interferences. It seems obvious that the use of QDs was inspired by the undoubted advantages of their applications in optical sensing systems including photochemical sensors [197,239,249,256,259]. More common CdS QDs are also used as passive carriers for aptamers [208] or sources of Cd ion detection using DPV [181,182,198]. QDs are mostly combined with other modifiers such as MWCNTs that provide either support for QDs implementation or their electric wiring with the electrode. Considering the high sensitivity of electroluminescence and photocurrent measurements, such components tend to increase their involvement in the aptasensor assembly. Regarding the signal measurement modes, there are two opposite trends that are directed to the broad use of biochemical amplification principles (see Section 5.3) and simplified detection schemes for point-on-demand use outside chemical laboratories. The first way is very effectively applied by long, multistep measurement protocols, which are hardly applicable in the field. The detection and quantification limits achieved are so small that do not have adequate tasks in food/drug analysis. Regarding simplified, one-step detection schemes and minimal sample treatment demanded in portable sensor design, the progress in electrochemical aptasensors for antibiotic determination is rather modest. Thus, a portable thin-film Au electrode modified with carboxylated MWCNTs and aptamer against oxytetracycline was used for on-site detection of the drug in chicken [222]. Certainly, many other aptasensors described in Table 2 can be further applied in portable sensing, especially those utilizing screen-printed electrodes [177,181,182,190], but some additional efforts are required to establish the robustness of appropriate sensors and satisfactory metrological characteristics of the response. The stability of immobilized aptamers and the multiuse of aptasensors are not considered presently as indispensable requirements of portable devices because their single-use application is more important from the point of view of biological contamination with previous samples tested and potential false results followed from such contamination. The in-line analysis of antibiotics based on electrochemical aptasensors is another challenge, which is not yet reached with conventional techniques. Probably the most critical problem of such devices is related to the necessity of incubation of the aptamer with target drugs and the equilibrium of aptamer–drug interaction shifted toward the product. This makes it impossible to follow changes in the concentrations of antibiotics on a real-time scale. Although the examples of on-site electrochemical analysis of other environmental toxicants such as pesticides are known [270], there are no prototypes of electrochemical aptasensors for drug residue detection. Probably, this might be possible for one-step detection schemes coupled with the displacement of analytes in the complex proposed for some other small molecules [271]. An alternative approach involves the electrochemical determination of microbial contamination biomarkers such as pyocyanin indirectly related to the advanced antibiotics resistance of some pathogens [272].

A review of electrochemical aptasensors for antibiotic determination confirms the advantages of electrochemical detection frequently mentioned for the detection of biochemical interaction with specific receptors. They involve rather simple design of transducers and measurement devices, a well-elaborated theory of the signal, compatibility with most bioreceptors including aptamers, and a broad range of conditions for biosensor functioning. Electrochemical aptasensors are rather simply adapted for use by unqualified persons and do not require sophisticated sample treatment or expensive reagents. Meanwhile, it should be clearly noted that not all analytes can be detected in electrochemical mode. Implementation of redox labels and the use of diffusionally free indicators compensates for this drawback but makes complicated measurement protocols and elongates the time of the measurement. The problems of in-line and continuous monitoring of affinity interactions, including aptamer–antibiotic interactions, have been mentioned above. Label-free techniques offer special attention to the control of the mass transfer at the electrode interface and can cause false-positive results yielded from the transfer of the sample components to the aptamer location.

It should be mentioned that the sensitivity of the conventional methods is comparable with those of the electrochemical aptasensors. However, there are several advantages of electrochemical aptasensors, as summarized in Table 3.

## 6. Conclusions

In this review, a summary of the most recent achievements in the development of electrochemical biosensors based on nucleic acid aptamers for the detection of antibiotics was provided. Most of the developed biosensors, especially those based on the nanomaterials, display high sensitivity and selectivity, with limits of detection well below MRL. Despite the substantial achievements made, aptasensors are still immature with respect to finding practical applications in medical, food, or environmental laboratories. However, the advantages of aptasensors pointing to the fast and low-cost detection of antibiotic contamination in food are obvious; hence, their more widespread employment is to be soon expected. Concerning food analysis, most of the studies focused on the detection of antibiotics in milk have been performed without significant effort made in the prevention of the fouling of the sensing surface by several food components such as proteins or sugars. This is a rather challenging topic on which future research should be primarily focused. In addition, the selectivity and sensitivity of aptasensors can be further optimized using computational methods to improve aptamer sequences, as well as with the use of advanced, antifouling surface functionalization chemistries for aptamer immobilization. It is also desirable to optimize protocols for sensor regeneration, allowing the reuse of the developed aptasensors in the field, thus further reducing costs and waste.

## Figures and Tables

**Table 1:** The maximum residue limit (MRL) for selected antibiotics in cow’s milk [13,14].

Substance	MRL [µg/kg]	Substance	MRL [µg/kg]
*β-lactam antibiotics*		*Sulfonamide antibiotics*	
Penicillin G	4	Sulfamethazine	100
Amoxicillin	4	Sulfadiazine	100
Ampicillin	4	Sulfadimethocxine	100
Oxacillin	30	Sulfaquinoxaline	100
Cloxacilliin	30	Sulfapyridine	100
Dicloxacillin	30	Sulfamethoxypyridazine	100
Nafcillin	30	Sulfamerazine	100
Cephquinome	20	Sulfachloropyridazine	100
Ceftiofur	100	*Tetracycline antibiotics*	
Cefazolin	50	Tetracycline	100
Cefacetrile	125	Oxytetracycline	100
Cefaperazone	50	Chlorotetracycline	100
Cephapirin	60	Doxycycline	0
		*Aminoglycoside antibiotics*	
		Kanamycin	0.4

**Table 2:** Electrochemical aptasensors for antibiotic determination (2018–2022).

Aptamer	Surface Layer	Detection Mode	Real Sample	Dynamic Range, LOD	Ref.
**Kanamycin**					
5′-TGG TGG GGG TTG AGG CTA AGC CG-3′	Au electrode covered with auxiliary DNA hairpin	Target-triggered cascade enzymatic recycling couple with DNAzyme amplification initiated by aptamer–analyte interaction. Hemin is used as a redox probe and H_2_O_2_ reduction current as a signal measured by DPV	Milk	1 pM–10 nM, 0.5 pM	[175]
5′-NH_2_-(CH_2_)_6_-TGG GGG TTG AGG CTA AGC CGA C-3′	Bare GCE	Zr MOF (UiO-66-NH_2_) saturated with Pb^2+^ ions as label of auxiliary DNA hybridized with the aptamer on the surface of magnetic beads. Target interaction released it, and Pb^2+^ was determined via DPV in stripping voltammetry mode	Milk	0.002 nM–100 nM, 0.16 pM	[176]
5′-TGG GGG TTG AGG CTA AGC CGA-3′-NH_2_	Screen-printed graphite electrode modified with aptamer via diazonium salt grafting and carbodiimide binding	EIS measurements with [Fe(CN)_6_]^3−/4−^ redox probe	Milk	1.2–75 ng/mL, 0.11 ng/mL	[177]
5′-TGG GGG TTG AGG CTA AGC CGA CTC AGA GAT CCA TAT GGA ACC CCC A-3′	Au electrode with covalently attached hairpin DNA containing aptamer sequence (underlined)	Interaction with analyte triggers strand displacement amplification with hybridization chain reaction, followed by intercalation of the MB molecules, which signal is determined by DPV	Milk	0.05–200 pM, 36 fM	[178]
5′-TCA GCG GGG AGG AAG AGA TGG GGG TTG AGG CTA AGC CGA GGA GTA-3′	Au bars modified with hybridized DNA probe and auxiliary DNA sequence initiating recycling hybridization synthesis. Aptamer sequence is underlined	Interaction with analyte-activated, toehold-mediated strand displacement reaction increased the MB quantities accumulated in double-stranded products. DPV signal increased with analyte concentration	Milk	0.05 pM–50.0 nM, 16.0 fM	[179]
5′-HS-(CH_2_)_6_-TGG GGG TTG AGG CTA AGC CGA CCG TAA-3′	GCE modified with hybridized DNA probe and auxiliary DNA sequence initiating recycling hybridization synthesis with transfer of the MOF-labeled sequences and release of MB. Aptamer sequence is underlined	Interaction with analyte-activated strand displacement reaction resulted in release of MB recorded with SWV	Milk, fish	0.1 pM–50 nM, 35 fM	[180]
5′-NH_2_-AGA TGG GGG TTG AGG CTA AGC CGA-3′	Screen-printed carbon electrodes modified with ordered mesoporous carbon fibers mixed with Au nanoparticles, followed by auxiliary DNA adsorption and their hybridization with CdS particles bearing aptamer	Interaction with analyte resulted in release of CdS containing aggregates, followed by DPV measurement of Cd^2+^ signal with stripping voltammetry	Milk	0.1–1000 nM, 87.3 pM	[181]
5′-NH_2_-AGA TGG GGG TTG AGG CTA AGC CGA-3′	Screen-printed electrode modified MWCNTs and Au nanoparticles bearing aptamer labeled with CdS nanochains	Interaction with analyte resulted in release of CdS containing aggregates, followed by DPV measurement of Cd^2+^ signal with stripping voltammetry	Milk	0.1–100 nM, 74.50 pM	[182]
5′-SH-(CH_2_)_6_-TCG GCT TAG CCT CAA CCC CCA-3′	Au electrode covered with capturing DNA hybridized with biotinylated aptamer and saturated with MB.	Interaction with analyte removes aptamer from the electrode interface; Au nanoparticles modified with streptavidin and HRP are added. DPV measurement of the signal related to the enzymatic MB oxidation after addition of H_2_O_2_.	Milk	2.0 pg/mL–100 ng/mL, 0.88 pg/mL	[183]
5′-ACT TCT CGC AAG ATG GGG GTT GAG GCT AAG CCG AAT ACT CCA GT-Fc-3′	Au electrode covered with mesoporous carbon-biotinylated Au nanoparticles and streptavidin conjugate of auxiliary DNA	DPV measurement of the Fc signal increased with the analyte concentration due to hybridization of the analyte–aptamer complex with auxiliary DNA on the electrode surface	Milk	0.1 nM–4 μM, 35.69 pM	[159]
5′-TGG GGG TTG AGG CTA AGC CGA GTC AC-3′	Au electrode covered with assistant DNA probe partially hybridized with aptamer	Interaction with analyte removes aptamer from the electrode and signaling DNA probe bearing MB is attached. DPV measurement of the MB signal increased with analyte concentration	Human serum, river water, milk	10 pM–1.0 μM, 3.3 pM	[184]
5′-TGG GGG TTG AGG CTA AGC CGA-3′	Au electrode modified with partially complementary aptamer and auxiliary DNA bearing MB label	SWV of the MB signal increased after analyte binding and conformational changes in the labeled DNA sequence (DNA folding). Alternatively, labeled DNA sequence displaces aptamer (labeled sequence shift)	Milk, tap water	10.0 nM–10.0 μM, 3.0 nM (DNA folding) 200.0 pM–1.0 μM, 60.0 pM (labeled sequence shift)	[185]
5′-AGA TGG GGG TTG AGG CTA AGC CGA-3′	Au electrode modified with binding DNA bonded to the aptamer labeled with thionine saturated Au@Pt core–shell nanoparticles	When aptamer is bonded to analyte, signaling DNA labeled with Au@Pt core–shell nanoparticles and thionine replaced that in the complex; DPV signal of thionine increased with analyte concentration	Chicken	1 pM–1 μM, 0.16 pM	[186]
5′-AGA TCC TAG GAG GCA CAT GTA AGA GTA GAT GGG GGT TGA GGC TAA GCC GAT AGC TA-3′	Au bars for accumulation of the apoferritin particles loaded with Pb^2+^ ions bonded to the specific aptamer	Release of Pb^2+^ ions in acidic media and their detection by SWV stripping voltammetry	Milk, fish	0.05 pM–50 nM, 18 fM	[187]
5′-TGG GGG TTG AGG CTA AGC CGA CGC GCG CG-(CH_2_)_6_-3′	Au bars for accumulation of the aptamer–MOF bearing F^−^ ions	Release of F^−^ ions measured potentiometrically with ISE	Milk, fish, urine, blood serum	1.0–200 nM, 0.35 nM	[188]
3′-NH_2_-(CH_2_)_6_-TCT GGG GGT TGA GGC TAA GCC GAC AG-5′	GCE covered with Zr containing MOF (UiO-66-NH_2_), melamine–cyanuric acid COF and MWCNT@rGO COF; DNA–aptamer hybrid is attached to the modifier	SWV of the MB signal reducing after removal of the aptamer–analyte complex from the sensor surface	Fish meat, milk	25–900 nM, 13 nM	[189]
5′-NH_2_-(CH_2_)_6_-AGA TGG GGG TTG AGG CTA AGC CGA-3′	Pt electrode covered with Ag nanoparticles and aptamer	ECL signal of luminal H_2_O_2_ system decreased with increased analyte concentration due to luminescence quenching by aptamer–analyte complexation	Milk	0.5–100 ng/mL, 0.06 ng/mL	[190]
5′-TAG CCT TTT TTT GGG GGT TGA GGC TAA GCC GAC-3′	Au electrode modified with thiolated oligonucleotides involved in binding of auxiliary DNA labeled with MB, which yield is activated by binding target analyte with aptamer sequence of the DNA hairpin (underlined part of the sequence)	DPV signal of the MB accumulated in the surface layer of ds-DNA formed in the bipedal DNA machine activated by aptamer–analyte interaction	Drinking water	10 fM–100 pM, 7.1 fM	[191]
5′-SH-AGA TGG GGG TTG AGG CTA AGC CGA-3′	Four-channel, screen-printed carbon electrode modified with rGO and dendritic Au nanostructures with attached thiolated aptamer	Potentiometric signal against electrode covered with polyA oligonucleotide	Milk	10 pM–1 μM, 5.24 pM	[192]
5′- (COOH)-TGG GGG TTG AGG CTA AGC CGA AAA AAA A-3′	GCE covered with rGO–Au nanoparticles in chitosan film and the MIP obtained from electropolymerization of 3-aminophenylboronic acid in the presence of the analyte	DPV signal of ferrocene attached to the Au@Fe_3_O_4_ particles covered with thiolated cyclodextrin and aptamer. Signal detection after accumulation of the particles on the analyte adsorbed on the MIP layer	Milk, tap water, artesian water, groundwater	10–500 nM, 1.87 nM	[193]
5′-TGG GGG TTG AGG CTA AGC CGA CCC CCC CCC CCC CCC-3′	GCE covered with MWCNTs and GO containing adsorbed aptamer.	DPV of the [Fe(CN)_6_]^3−/4−^ redox probe.	Milk	0.05 pM–100 nM, 0.0476 pM	[194]
3′-NH_2_-TGG GGG TTG AGG CTA AGC CGA-C-5′	GCE covered with carbon black and Calix [4] arene-bearing lactic fragments, aminated aptamer covalently attached via carbodiimide binding	EIS measurements with [Fe(CN)_6_]^3−/4−^ redox probe	Milk, yogurt	0.7–50 nM, 0.3 nM	[195]
5′-AGA TGG GGG TTG AGG CTA AGC CGA-3′	GCE modified with perylene derivative and aptamer adsorbed on Au@Cu_2_O heterostructures	ECL signal of S_2_O_8_^2−^ reduction increased with the analyte concentration due to removal of aptamer from the sensor surface	Milk	0.1 pM–10 nM, 0.042 pM	[196]
5′-SH-(CH_2_)_6_-TGG GGG TTG AGG CTA AGC CGA-3′	ITO glass modified with CuO/Pd nanocomposite and thiolated aptamer hybridized with auxiliary DNA conjugated with CdS QD	Photoelectrochemical signal recorded after aptamer–analyte interaction and auxiliary DNA removal	Milk	0.1–500 nM, 20 pM	[197]
5′-Bio-ACC GCG GGG UUG CGG ACC GGG AGC UCC AGC-NH_2_-3′	Au electrode modified with Au nanoparticles and CdS-aptamer conjugated via streptavidin–biotin binding	DPV, increase in the ferricyanide peak due to removal of aptamer–analyte complex from the surface	Milk	1–400 nM; 0.12 nM	[198]
5′-TGG GGG TTG AGG CTA AGC CGA GGA GTA-3′	Au electrode covered with auxiliary DNA complementary to DNA fragment of a hairpin DNA coupled to the aptamer	DPV signal of HRP reaction with MB and H_2_O_2_. Enzyme is coupled to the auxiliary DNA on the electrode after amplification of the aptamer–hairpin DNA structures in the presence of exonuclease EXO-1	Milk, honey	0.05 pg/mL–10 ng/mL, 9.1 fg/mL	[199]
**Tobramycin**					
5′-HS-GGCA CGAG GUUU AGCU ACAC UCGU GCC-3′	Graphene-based field-effect transistor with aptamer in the channel area.	Drain current.	Water	0.3 nM	[200]
5′-AAA AAA GAC TAG GCA CTA GTC AAA AAA CCC CGA TCC TAG TCT TTC CC-3′	Au electrode modified with signaling DNA.	Analyte initiates multiple recycling via strand displacement DNA polymerization; final product interacts with signaling DNA, and the quantity of hybridized structure is determined with the current of [Ru(NH_3_)_6_]^3+^ redox probe in DPV mode	Milk, water	10–200 nM, 5.13 nM	[201]
5′-ACU UGG UUU AGG UAA UGA GU-3′	Au electrode modified with calcinated CeO_2_/CuO_x_ MOF and aptamer	EIS measurements with [Fe(CN)_6_]^3−/4−^ redox probe.	Human serum, milk	0.01 pg/ mL–10 ng/ mL, 2.0 fg/ mL	[202]
5′-Bio-GGC ACG AGG UUU AGC UAC ACU CGU GCC NH_2_-3′	Au electrode modified with Au nanoparticles and PbS–aptamer conjugated via streptavidin–biotin binding	DPV, increase in the ferricyanide peak due to removal of aptamer–analyte complex from the surface	Milk	1–10,000 nM, 0.49 nM	[198]
5′-GGG ACT TGG TTT AGG TAA TGA GTC CC-3′	GCE modified with poly(ethylene imine), Fe MOF, and Au nanoparticles with thiolated binding DNA complementary to the aptamer	Aptamer competitively reacts either with analyte or binding DNA. After that, free binding DNA is hybridized with signaling DNA bearing MB. DPV signal of MB increases with the analyte concentration	Milk	100 pM–500 nM, 56 pM	[203]
5′-ACU UGG UUU AGG UAA UGA GU-3′	Au electrode modified with CoNi metal–covalent organic frameworks based on phthalocyanine tetra-amine and phenanthroline derivatives	EIS measurements with [Fe(CN)_6_]^3−/4−^ redox probe	Milk, chicken eggs	0.1 fg/mL–1 pg/mL, 0.07 fg/mL	[204]
**Streptomycin**					
5′-GTT TGT GTA TTA CAG TTA TGT TAC CCT CAT TTT TCT GAA-C-3′	Au electrode covered with thiolated β-cyclodextrin and aptamer	EIS measurements with [Fe(CN)_6_]^3−/4−^ redox probe	Milk, tap and lake water, bacteria culture medium	0.01–100 nM, 0.008 nM	[205]
5′-TAG GGA ATT CGT CGA CGG ATC CGG GGT CTG GTG TTC TGC TTT GTT CTG TCG GGT CGT CTG CAG GTC GAC GCA TGC GCC G-SH-3′	GCE covered with graphene QDs modified with amino and thiol groups, Au nanoparticles, and Ag nanoparticles with attached aptamer	EIS measurements with [Fe(CN)_6_]^3−/4−^ redox probe	Human serum	0.01–812.21 pg/mL, 0.0033 pg/mL	[206]
5′-TAG GGA ATT CGT CGA CGG ATC CGG GGT CTG GTG TTC TGC TTT GTT CTG TCG GGT CGT CTG CAG GTC GAC GCA TGC GCC G-SH-3′.	GCE covered with thiolated graphene QDs, and Au nanoparticles with attached aptamer	EIS measurements with [Fe(CN)_6_]^3−/4−^ redox probe	Human serum	0.1–700 pg/mL, 0.033 pg/mL	[207]
5′-NH_2_-GGG GTC TGG TGT TCT GCT TTG TTC TGT CGG GTC GT-3′	Screen-printed carbon electrodes modified with ordered mesoporous carbon fibers mixed with Au nanoparticles, followed by auxiliary DNA adsorption and their hybridization with PbS particles bearing aptamer	Interaction with analyte resulted in release of PbS containing aggregates followed by DPV. measurement of Pb^2+^ signal with stripping voltammetry	Milk	0.1–1000 nM, 45.0 pM	[181]
5′-NH_2_-GGG GTC TGG TGT TCT GCT TTG TTC TGT CGG GTC GT-3′	Screen-printed electrode modified MWCNTs and Au nanoparticles bearing aptamer labeled with PbS nanochains	Interaction with analyte resulted in release of PbS containing aggregates, followed by DPV measurement of Pb^2+^ signal with stripping voltammetry	Milk	0.1–100 nM, 36.45 pM	[182]
5′-TAG GGA ATT CGT CGA CGG ATC CGG GGT CTG GTG TTC TGC TTT GTT CTG TCG GGT CGT CTG CAG GTC GAC GCA TGC GCC G-SH-3′	GCE covered with thiourea capped CdS QDs and covalently attached aptamer	EIS measurements with [Fe(CN)_6_]^3−/4−^ redox probe	Milk, blood serum	1 fg/mL–1.111 pg/mL, 1.111 pg/mL–11.1111 ng/mL, 0.35 fg/mL	[208]
5′-TAG GGA ATT CGT CGA CGG ATC CGG GGT CTG GTG TTC TGC TTT GTT CTG TCG GGT CGT CTG CAG GTC GAC GCA TGC GCC G-SH-3′.	Au electrode modified with mesoporous silica film and aptamer	DPV and EIS measurements with [Fe(CN)_6_]^3−/4−^ redox probe	Milk, blood serum	1 fg/mL–6.2 ng/mL, 0.33 fg/mL	[209]
5′-GGG GTC TGG TGT TCT GCT TTG TTC TGT CGG GTC GT-3′	Screen-printed carbon electrode modified with Au nanoparticles with attached auxiliary DNA hybridized with aptamer	Interaction with analyte initiated removal of aptamer from the electrode surface by endonuclease-assisted cleavage. After that, MOF-based biobarcode was attached, and its quantity was determined via DPV signal of [Ru(NH_3_)_6_]^3+^ redox probe	Milk	0.005–150 ng/mL, 2.6 pg/mL	[210]
5′-SH-TAG GGA ATT CGT CGA CGG ATC CGG GGT CTG GTG TTC TGC TTT GTT CTG TCG GGT CGT CTG CAG GTC GAC GCA TGC GCC-G-3′	Four-channel screen-printed carbon electrode modified with rGO and dendritic Au nanostructures with attached thiolated aptamer	Potentiometric signal against electrode covered with polyA oligonucleotide	Milk	10 pM–10 μM, 9.66 pM	[191]
**Tetracycline**					
5′-NH_2_-(CH_2_)_6_-CGT ACG GAA TTC GCT AGC CCC CCG GCA GGC CAC GGC TTG GGT TGG TCC CAC TGC GCG TGG ATC CGA GCT CCA CGT G-3′	Screen-printed, carbon electrode modified with N-octylpyridinium hexafluorophosphate and Fe_3_O_4_ dispersed in chitosan. Aptamer attached to the modifier	Peak currents of the [Fe(CN)_6_]^3−/4−^ redox probe were measured using cyclic voltammetry	Milk	1.0 nM–10 mM, 1.0 nM	[211]
5′-CGT ACG GAA TTC GCT AGC CCC CCG GCA GGC CAC GGC TTG GGT TGG TCC CAC TGC GCG TGG ATC CGA GCT CCA CGT G-3′	Screen-printed, carbon electrode covered with ordered mesoporous carbon–Fe_3_O_4_ composite and physically adsorbed aptamer	DPV signal of thionine as redox probe added to the aptasensor	-	5 nM–10 μM, 0.8 nM	[212]
5′-HS-CGT ACG GAA TTC GCT AGC CCC CCG GCA GGC CAC GGC TTG GGT TGG TCC CAC TGC GCG TGG ATC CGA GCT CCA CGT G-3′.	Au electrode modified with the monolayer of thiolated aptamer	MWCNTs with loaded Au nanoparticles were modified with the auxiliary DNA complementary to aptamer and saturated with thionine. In the interaction with analyte, it left the electrode, and DPV signal of thionine decreased with increased analyte concentration	Chicken	0.1 nM–1 μM, 0.06 nM	[213]
5′-SH-(CH_2_)_6_-CGT ACG GAA TTC GCT AGC CCC CCG GCA GGC CAC GGC TTG GGT TGG TCC CAC TGC GCG TGG ATC CGA GCT CCA CGT G-3′	GCE modified with the MoS_2_–TiO_2_ nanocomposite and aptamer	First, aptasensor was treated with analyte solution. Then, signaling biotinylated DNA interacted with aptamer molecules that were not involved in the reaction with analyte. After that, avidin–HRP conjugate was added, and the enzyme activity quantified by DPV signal of the hydroquinone–benzoquinone redox pair interacted with HRP in the presence of H_2_O_2_	Milk	0.15 nM–6.0 μM, 0.05 nM	[214]
5′-CGT ACG GAA TTC GCT AGC CCC CCG GCA GGC CAC GGC TTG GGT TGG TCC CAC TGC GCG TGG ATC CGA GCT CCA CGT G-3′	Screen-printed, carbon electrode grafted with diazonium salt. Aptamer attached to carboxylic groups by carbodiimide binding	LSV with the [Fe(CN)_6_]^3−/4−^ redox probe	Lake water	0.05–20 μg/L 0.035 μg/L	[215]
5′-TCT CTC GGT GGT GTC CTC TCT-3′	Au electrode covered with thiolated β-cyclodextrin	Triple helix aptamer probes interact with analyte by releasing trigger probe initiating catalytic hybridization of two hairpins bearing Fc units. Afterward, exonuclease III demolished hybridization products and released Fc accumulated in the cyclodextrin moiety. DPV signal of Fc increased with the analyte concentration	Milk	0.2 nM–100 nM, 0.13 nM	[216]
5′-CGT ACG GAA TTC GCT AGC CCC CCG GCA GGC CAC GGC TTG GGT TGG TCC CAC TGC GCG TGG ATC CGA GCT CCA CGT G-3′ and 5′-GTT TGT GTA TTA CAG TTA TGT TAC CCT CAT TTT TCT GAA-C-3′	Screen-printed Au electrode modified with equimolar mixture of two thiolated aptamers	SWV measurements with the [Fe(CN)_6_]^3−/4−^ redox probe	Honey	0.01–1000 ng/mL, 0.0073 ng/mL	[135]
5′-SH-CCC CCG GCA GGC CAC GGC TTG GGT TGG TCC CAC TGC GCG-3′	Pre-oxidized pencil graphite electrode covered with magnetic nanoparticles with loaded Au layer and attached thiolated aptamer	EIS measurements with [Fe(CN)_6_]^3−/4−^ redox probe	Milk of cow, sheep, goat, and water buffalo	1.0 pM–1.0 μM, 0.03 pM	[217]
5′-SH-CGT ACG GAA TTC GCT AGC CCC CCG GCA GGC CAC GGC TTG GGT TGG TCC CAC TGC GCG TGG ATC CGA GCT CCA CGT-G-3′	Screen-printed carbon electrode covered with carbonized Fe-based MOF, Au nanoparticles with attached thiolated aptamer	EIS measurements with [Fe(CN)_6_]^3−/4−^ redox probe	Tap water, lake water	0.1 nM–100 μM, 0.01 nM	[218]
5′-NH_2_-(CH_2_)_6_-CGT ACG GAA TTC GCT AGC CCC CCG GCA GGC CAC GGC TTG GGT TGG TCC CAC TGC GCG TGG ATC CGA GCT CCA CGT-G-3′	ITO electrode covered with CdTe–BiOBr heterojunction, chitosan, and physically adsorbed aptamer	Photocurrent measured at −0.2 V	Soil	10–1500 pM, 9.25 pM	[219]
5′ -SH-CGT ACG GAA TTC GCT AGC CCC CCG GCA GGC CAC GGC TTG GGT TGG-TCC-CAC-TGC- GCG-TGG ATC CGA GCT CCA CGT-G-3′	GCE covered with composite of WO_3_ and MWCNTs followed by electrodeposition of Au nanoparticles and attachment of thiolated aptamer	EIS measurements with [Fe(CN)_6_]^3−/4−^ redox probe	Tap, pond, and river water, milk, honey, black tea	0.1–100 nM, 0.048 nM	[220]
**Oxytetracycline**					
5′-CGT ACG GAA TTC GCT AGC CGA GGC ACA GTC GCT GGT GCC TAC CTG GTT GCC GTT GTG TGG ATC CGA GCT CCA CGT-G-3′	Au electrode covered with Ce-based MOF obtained from melamine and cyanuric acid and physically adsorbed aptamer	EIS measurements with [Fe(CN)_6_]^3−/4−^ redox probe	Milk, urine, river water	0.1–0.5 ng/ mL, 17.4 fg/ mL	[221]
5′-GGA ATT CGC TAG CAC GTT GAC GCT GGT GCC CGG TTG TGG TGC GAG TGT TGT GTG GAT CCG AGC TCC ACG-TG-3′	Resistive sensor with semiconductive SWCNT modified with aptamer	Measurement of the resistance shift after hybridization of excessive aptamer remained free after the contact with analyte solution	Real wastewater samples	10–75 μg/L, 1.125 μg/L	[143]
5′-SH-GGA ATT CGC TAG CAC GTT GAC GCT GGT GCC CGG TTG TGG TGC GAG TGT TGT GTG GAT CCG AGC TCC ACG TG-3′	Thin-film Au electrode modified with monolayer of thiolated aptamer.	Carboxylated MWCNTs were loaded with Au nanoparticles and thionine and modified with auxiliary DNA hybridized with the aptamer attached to the electrode. Interaction with analyte released MWCNT–DNA composite from the electrode and decreases the thionine current measured by DPV	Chicken	1 × 10^−13^–1 × 10^−5^ g/mL, 3.1 × 10^−14^ g/mL	[222]
5′-GGA ATT CGC TAG CAC GTT GAC GCT GGT GCC CGG TTG TGG TGC GAG TGT TGT GTG GAT CCC GAG CTC CAC GTG-3′	ITO glass electrode modified with Co_3_O_4_/g-C_3_N_4_ heterojunction and aptamer physically adsorbed on the layer	Photocurrent measurement	Water samples	0.01–500 nM, 3.5 pM	[223]
5′-NH_2_-CGT ACG GAA TTC GCT AGC GGG CGG GGG TGC TGG GGG AAT GGA GTG CTG CGT GCT GCG GGG ATC CGA GCT CCA CGT-G-3′.	ITO glass electrode modified with Bi/BiVO_4_/g-C_3_N_4_ heterojunction and aptamer attached by glutaraldehyde binding	Photocurrent measurement	Milk	0.01–1000 nM, 3.3 pM	[224]
5′-NH_2_-(CH_2_)_6_-GGA ATT CGC TAG CAC GTT GAC GCT GGT GCC CGG TTG TGG TGC GAG TGT TGT GTG GAT CCG AGC TCC ACG-TG-3′	Au electrode modified with porous organic framework synthesized from 1,3,5-tris-bromomethyl-2,4,6-trimethylbenzene and tetraphenylethylene with electrodeposited Au nanoparticles and aptamer	EIS measurements with [Fe(CN)_6_]^3−/4−^ redox probe	Milk, human serum, urine, river water	1.0 × 10^−5^– 1.0 ng/mL, 3.19 fg/mL	[225]
5′-NH_2_-GGA ATT CGC TAG CAC GTT GAC GCT GGT GCC CGG TTG TGG TGC GAG TGT TGT GTG GAT CCG AGC TCC ACG TG-3′	GCE grafted with diazonium salt, followed by aptamer attachment by carbodiimide binding	DPV measurements with the [Fe(CN)_6_]^3−/4−^ redox probe	Milk	1.0 × 10^−9^– 1.0 × 10^−4^ g/mL, 2.29×10^−10^ g/mL	[226]
5′-Fc-GGA ATT CGC TAG CAC GTT GAC GCT GGT GCC CGG TTG TGG TGC GAG TGT TGT GTG GAT CCG AGC TCC ACG TG-(CH_2_)_6_-SH-3′	Screen-printed carbon electrodes covered with a thin Au layer obtained electrochemically in different modes. Thiolated aptamer with terminal Fc group attached to Au by Au–S bonding	DPV measurement of Fc signal after analyte–aptamer binding and aptamer structure folding	Milk	50 nM–1.2 μM, 8.7 nM	[227]
**Ciprofloxacin**					
5′-ATA CCA GCT TAT TCA ATT GCA GGG TAT CTG AGG CTT GAT CTA CTA AAT GTC GTG GGG CAT TGC TAT TGG CGT TGA TAC GTA CAA TCG TAA TCA GTT AG-3′	Au electrode covered with the COF based on 1,3,5-tris(4-aminophenyl)benzene and 2,5- dimethoxyterephaldehyde with electrodeposited Au nanoparticles and attached aptamer	EIS measurements with [Fe(CN)_6_]^3−/4−^ redox probe	Milk, human serum, river water, urine	3.02 × 10^–5^–1.51 nM, 7.06 fM	[228]
5′-NH_2_-ATA CCA GCT TAT TCA ATT GCA GGG TAT CTG AGG CTT GAT CTA CTA AAT GTC GTG GGG CAT TGC TAT TGG CGT TGA TAC GTA CAA TCG TAA TCA GTT AG-3′	GCE covered with rGO, aminated dendrimer PAMAM and Au nanoparticles, aptamer reduced graphene oxide	DPV measurements with [Fe(CN)_6_]^3−/4−^ redox probe	Milk	1 nM–1 mM, 1 nM	[229]
5′-NH_2_-ATA-CCA-GCT-TAT- TCA-ATT-GCA-GGG-TAT-CTG-AGG-CTT-GAT-CTA-CTA-AAT-GTC-GTG-GGG-CAT-TGC-TAT-TGG-CGT-TGA-TAC-GTA-CAA-TCG-TAA-TCA-GTT-AG-3′	ITO glass electrode modified with rGO doped with Bi^3+^/black anatase TiO_2_ and adsorbed aptamer	Anodic photocurrent	Milk	0.01–1000 ng/mL, 3.3 pg/mL	[230]
**Ofloxacin**					
5′-SH-ATA CCA GCT TAT TCA ATT AGT TGT GTA TTG AGG TTT GAT CTA GGC ATA GTC AAC AGA GCA CGA TCG ATC TGG CTT GTT CTA CAA TCG TAA TCA GTT AG-3′.	Ti foils anodized and covered with Ag_2_S and self-polymerized polydopamine film. Aptamer immobilized via physical adsorption	Photocurrent measurement	Milk	5.0 pM–100 nM, 0.75 pM	[231]
5′-NH_2_-(CH_2_)_6_-ATA CCA GCT TAT TCA ATT AGT TGT GTA TTG AGG TTT GAT CTA GGC ATA GTC AAC AGA GCA CGA TCG ATC TGG CTT GTT CTA CAA TCG TAA TCA GTT AG-3′.	ITO glass electrode modified with [Ru(bpy)_3_]^2+^@Ce-UiO-66/Mn:Bi_2_S_3_ heterojunction followed by immobilization of aptamer in chitosan matrix by glutaraldehyde linking	[Ru(bpy)_3_]^2+^ photocatalytic current	Tap water, lake water	0.01−100 nM, 6 pM	[232]
**Enrofloxacin**					
5′-CCC ATC AGG GGG CTA GGC TAA CAC GGT TCG GCT CTC TGA GCC CGG GTT ATT TCA GGG GGA-3′	Au electrode modified with the COF based on 1,3,6,8-tetrakis(4-formylphenyl)pyrene and melamine, aptamer physically adsorbed on the surface	EIS measurements with [Fe(CN)_6_]^3−/4−^ redox probe	Human serum	0.01 pg/mL–2 ng/mL, 6.07 fg/mL	[233]
5′-CCC ATC AGG GGG CTA GGC TAA CAC GGT TCG GCT CTC TGA GCC CGG GTT ATT TCA GGG GGA-3′	Au electrode modified with Co/Fe polyphtalocyanine and physically adsorbed aptamer	EIS measurements with [Fe(CN)_6_]^3−/4−^ redox probe	River water, milk, pork	0.1 fg/mL–100 pg/mL, 0.06 fg/mL	[234]
5′-NH_2_-CCC ATC AGG GGG CTA GGC TAA CAC GGT TCG GCT CTC TGA GCC CGG GTT ATT TCA GGG GGA-3′	ITO glass electrode modified with rGO doped with Bi^3+^/black anatase TiO_2_ and adsorbed aptamer	Anodic photocurrent	Milk	0.01–10,000 ng/mL, 3.3 pg/mL	[230]
**Sulfaquinoxaline**					
5′-GTA ACC CTG CCA CAT CCA ACC CCC ATG TTG GCT CTT AC-3′	Au electrode modified with poly(ethylene imine)–CoSe_2_ and Zr-based MOF UiO-66–NH_2_ with chemically synthesized Au–Pd nanoparticles. On them, thiolated auxiliary DNA is covalently attached and hybridized with the aptamer	Interaction with analyte results in release of the aptamer from the electrode interface. Treatment with the RecJf exonuclease reduced the length of auxiliary DNA. After that, DPV signal of [Fe(CN)_6_]^3−/4−^ redox probe was measured	Pork	1 pg/mL–100 ng/mL, 0.547 pg/mL	[235]
**Sulfamethazine**					
5′-SH-TTA GCT TAT GCG TTG GCC GGG ATA AGG ATC CAG CCG TTG TAG ATT TGC GTT CTA ACT CTC-Fc-3′	Au electrode modified with Ag@Au core–shell nanoparticles and thiolated aptamer	DPV signal of Fc label	Pork	0.1–50 ng/mL, 0.033 ng/mL	[236]
5′-NH_2_-TTA GCT TAT GCG TTG GCC GGG ATA AGG ATC CAG CCG TTG TAG ATT TGC GTT CTA ACT CTC-3′	GCE covered with WS_2_–carbon QDs, followed by aptamer adsorption	After interaction with the analyte, poly(ethylene imine) was added and the current of the [Ru(NH_3_)_6_]^3+^ redox probe was measured in DPV mode	Pork	10 pM–1.0 µM, 4.0 pM	[237]
**Sulfadimethoxine**					
5′-GAG GGC AAC GAG TGT TTA TAG-3′	Au electrode covered with thiolated capturing DNA partially hybridized with aptamer	Interaction with analyte removed aptamer–analyte complex from the electrode interface. SWV signal of the [Fe(CN)_6_]^3−/4−^ redox probe	Lake water	1 nM–1 mM, 1 nM	[238]
5′-GAG GGC AAC GAG TGT TTA TAG A-3′	ITO glass Electrode modified with GO, g-C_3_N_4_ QDs, and aptamer	Photocurrent measurements	Tap, lake, and wastewater	0.5–80 nM, 0.1 nM	[239]
5′-SH-GAG GGC AAC GAG TGT TTA TAG A-3′	Pencil graphite electrode modified with rGO, electrodeposited Au nanoparticles, and thiolated aptamer	EIS signal of the [Fe(CN)_6_]^3−/4−^ redox probe	Fish, chicken, beef	1.0 fM–10 μM, 0.37 fM	[240]
**Penicillin**					
5′-SH-TTA GTT GGG GTT CAG TTG G-3′	Pencil graphite electrode modified with rGO and electrodeposited Au nanoparticles with thiolated aptamer	EIS signal of the [Fe(CN)_6_]^3−/4−^ redox probe	Milk of cow, sheep, goat and water buffalo	1.0 fM–10 μM, 0.8 fM	[241]
5′-NH_2_-CTG AAT TGG ATC TCT CTT CTT GAG CGA TCT CCA CA-3′	Au electrode covered with Ag-based COF with immobilized aptamer	EIS signal of the [Fe(CN)_6_]^3−/4−^ redox probe	Milk	0.001–0.5 ng/mL, 0.849 pg/mL	[242]
5′-GGG TCT GAG GAG TGC GCG GTG CCA GTG AGT-3′	Au electrode bearing thiolated aptamer with terminal MB group	SWV signal of the MB as redox label	Buffer Milk	0.3 nM 1.7 nM	[243]
5′-SH-(CH_2_)_6_-CTG AAT TGG ATC TCT CTT CTT GAG CGA TCT CCA CA-3′	Electrospun carbon nanofiber mat covered with Au nanoparticles and attached thiolated aptamer	LSV signal of the [Fe(CN)_6_]^3−/4−^ redox probe	Milk	1–400 ng/mL, 0.6 ng/mL	[244]
**Ampicillin**					
5′-SH-TGG GGG TTG AGG CTA AGC CGA C-3	GCE covered Au nanoparticles and aptamer bonded via Au–S interaction	Auxiliary DNA complementary to aptamer is first attached to the Au nanoparticles and then involved in the reaction with ssDNA binding protein that blocked the electrode surface. In the reaction with analyte, blocking particles are released and the DPV signal of the [Fe(CN)_6_]^3−^ ions increased	Milk	1 pM–5 nM, 0.38 pM	[245]
5′-TTA GTT GGG GTT CAG TTG G-3′	Au electrode modified with the COF based on 1,3,6,8-tetrakis(4-formylphenyl)pyrene and melamine, aptamer physically adsorbed on the surface	EIS signal of the [Fe(CN)_6_]^3−/4−^ redox probe	Human serum	0.01 pg/mL–2 ng/mL, 6.07 fg/mL	[233]
5′-NH_2_-(CH_2_)_6_-TAG CTA TCG GCT TAG CCT CAA CCC CCA TCT ACT CTT ACA TGT GCC TCC TAG GAT CT-3′	Au bars for accumulation of the apoferritin particles loaded with Cd^2+^ ions bonded to the specific aptamer	Release of Cd^2+^ ions in acidic media and their detection by SWV stripping voltammetry	Milk, fish	0.05 pM–50 nM, 15 fM	[187]
5′-TTG ATC GCG GGC GGT TGT ATA GCG G-3′	GCE covered with the monolayer of auxiliary DNA complementary to the aptamer	Aptamer is first mixed with the analyte and then added to the electrode. In the absence of the analyte, hybrid part of the layer is digested by exonuclease. In the opposite case, DNA monolayer is protected. The removal of DNA from the surface is determined by DPV signal of the [Fe(CN)_6_]^3−/4−^ redox probe	Milk, water	0.1–100 nM, 0.032 nM	[246]
HS-TTT TTT TTG TGG GTA GGG GGG GGT TGG GGC GGC C-3′	Au electrode covered with DNA walker-locking probe and DNA track via Au–S binding	DNA walker and locking probe form the duplex protected from exonuclease. Enzymatic amplification is triggered by the analyte specifically bonded to aptamer (underlined part of the sequence). As a result of the cycle, hemin binding part of the sequence interacts with them and biocatalytic reaction of H_2_O_2_ reduction is detected in DPV mode	Milk	1 pM to 10 nM, 0.76 pM	[247]
5′–Bio–GCG GGC GGT TGT ATA GCG G–3′	Magnetic GCE	Magnetic beads modified with ampicillin are involved in the reaction with biotinylated aptamer. After reaction, HRP–streptavidin conjugate is attached and HRP detected by linear scan voltammetry using hydroquinone/benzoquinone pair	Milk	0.1 pM–10 nM, 0.1 pM	[248]
5′-NH_2_-(CH_2_)_6_-TTT TGC GGG CGG TTG TAT AGC GG-3′	ITO glass electrode modified with N-doped graphene QDs and AgBiS_2_ dual-sensitized Zn/Co bimetallic oxide dodecahedron. Aptamer covalently attached by carbodiimide binding	Photocurrent measurements	Tap and lake waters	0.5 pM–10 nM, 0.25 pM	[249]
5′-NH_2_-(CH_2_)_6_-TTT TGC GGG CGG TTG TAT AGC GG-3′	ITO glass electrode covered with In_2_O_3_-In_2_S suspension followed by immobilization of aptamer in chitosan matrix by glutaraldehyde binding	Photocurrent measurements	Lake water	0.001–300 ng/mL, 0.06 pg/mL	[250]
5′-TTA GTT GGG GTT CAG TTG G-3′	Au electrode covered with porous organic polymer prepared by the coupled polymerization of tetraphenylpyrene and dibromo-p-xylene	EIS signal of the [Fe(CN)_6_]^3−/4−^ redox probe	Milk, river water, human serum	1 × 10^–5^–5 ng/mL, 1.33 × 10^–6^ ng/mL	[251]
5′-NH_2_-(CH_2_)_6_-GCG GGC GGT TGT ATA GCG G-3′	Screen-printed carbon electrode modified with MoS_2_ nanospheres covered with electro-synthesized polypyrrole. Then, ethylene diamine was electrochemically attached to the polypyrrole and then 1,4-naphthoquinone and aptamer were covalently attached by carbodiimide binding	SWV signal of the naphtaquinone redox label	River water	LOD 0.28 pM	[252]
**Amoxicillin**					
5′- SH-TTA GTT GGG GTT CAG TTG G-3′	GCE modified with TiO_2_-g-C_3_N_4_@AuNPs and thiolated aptamer	EIS signal of the [Fe(CN)_6_]^3−/4−^ redox probe	Wastewater	0.5–3 nM, 0.2 nM	[253]
**Azlocillin**					
5′-CAG GAA GAC AAC TCC GAC TAG AAT TGA TAA TCA AGA ATT CGT CTG GGG GGA ATG TGC G-3′	Au electrode covered with cysteine monolayer and covalently attached aptamer	DPV signal of the [Fe(CN)_6_]^3−/4−^ redox probe	Tap and wastewaters	1 pg/mL–100 mg/mL, 1.2 pg/mL	[254]
**Lincomycin**					
5′-NH_2_-(CH_2_)_6_-CGC GTG ATG TGG TCG ATG CGA TAC GGT GAG TCG CGC CAC GGC TAC ACA CGT CTC AGC GA-3′	ITO glass electrode modified with TiO_2_ and hollow ZnIn_2_S_4_ nanocages	Photocurrent measurements	Milk	0.1 pM–0.1 nM, 0.084 pM	[255]
**Chloramphenicol**					
5′-ACT TCA GTG AGT TGT CCC ACG GTC GGC GAG TCG GTG GTA G-3′	FTO electrode modified with TiO_2_ nanorods, CdS:Eu^3+^ QDs, and aptamer	Photocurrent measurements	Milk	1.0 pM–3.0 nM, 0.36 pM	[256]
5′-AGC AGC ACA GAG GTC AGA TGC ACT CGG ACC CCA TTC TCC TTC CAT CCC TCA TCC GTC CAC CCT ATG CGT GCT ACC GTG AA-Fc-3′	ITO glass electrode covered with g-C_3_N_4_ nanosheets and aptamer	Photocurrent measurements	Water	1 pM–100 nM, 0.22 pM	[257]
5′-ACT TCA GTG AGT TGT CCC ACG GTC GGC GAG TCG GTG GTA G-3′	Au electrode with hairpin DNA immobilized via terminal SH group	Analyte interacts with aptamer, which structure is stabilized by Ag+ ions. Their release activates DNAzyme that catalyzed removal of the DNA hairpin from the surface. This deceased charge transfer resistance measured by SWV with [Fe(CN)_6_]^3−/4−^ redox probe	Milk	1 pg/ mL–40 ng/ mL, 0.4 pg/mL	[258]
5′-ACT TCA GTG AGT TGT CCC ACG GTC GGC GAG TCG GTG GTA G-3′	Anodized Ti foil covered with Au QDs aptamer	Photocurrent measurements	Honey	0.5–100 nM, 57.9 pM	[259]
5′-NH_2_-ACT TCA GTG AGT TGT CCC ACG GTC GGC GAG TCG GTG GTA G-3′	GCE covered with 3-amino ethylpyridine and Ag nanoparticles with thiolated aptamer. After saturation with analyte, resorcinol was polymerized on the surface to obtain MIPs.	Interaction of the MIP polymer with analyte was assessed with EIS signal of the [Fe(CN)_6_]^3−/4−^ redox probe	Milk	1.0 pM–1.0 nM, 0.3 pM	[260]
5′-SH-ACT TCA GTG AGT TGT CCC ACG GTC GGC-3′	Screen-printed, carbon electrode modified with Au and hairpin aptamer	The reaction with analyte results in conformational changes followed by the binding of signaling biotinylated aptamer and finally streptavidin–HRP conjugate. LSV/amperometric signal of tetramethylbenzidine as HRP substrate in the presence of H_2_O_2_	-	10 nM–10 μM, 1 nM	[261]
5′-NH_2_-ACT TCA GAG AGT TGT CCC ACG GTC GGC GAG TCG GTG GTA G-3′	GCE covered with GO modified with 3-aminopropyltriethoxysilan followed by deposition of chemically synthesized Ag nanoparticles and thiolated aptamer bonded via AG–S interaction	DPV signal of the [Fe(CN)_6_]^3−/4−^ redox probe	Milk, honey	1–100 ng/mL, 0.70 ng/mL	[262]
5′-SH (CH_2_)_6_-ACT TCA GTG AGT TGT CCC ACG GTC GGC GAG TCG GTG GTA G-3′	Au electrode covered with poly(ethylene imine)–rGO composite and electrodeposited Au nanoparticles with attached thiolated aptamer	EIS signal of the [Fe(CN)_6_]^3−/4−^ redox probe enhanced by binding of specific protein to free aptamer molecules	Chicken	5 pM–1 μM, 2.08 pM	[263]
5′-TGT GTA CTT CAG TGA GTT GTC CCA CGG TCG GCG AGT CGG TGG TAG-3′	Au electrode covered with composite of Au nanowires and g-C_3_N_4_ in PEI matrix followed by immobilization of thiolated auxiliary DNA and hybridization with an aptamer	Interaction with analyte removed aptamer from the duplex with auxiliary DNA. After that, Recjf exonuclease cleaves DNA strands, and [Fe(CN)_6_]^3−/4−^ DPV signal is measured	Milk	100 fM–1 μM, 2.96 fM	[264]
5′--NH_2_-(CH_2_)_6_-ACC TGG GGG AGT ATT GCG GAG GAA GGT-3′	Au electrode covered with UiO-66–NH_2_@COF particles based on 2-aminoterephthalic acid, 1,3,5-tris (4-aminophenyl)benzene, and 2,5-dimethoxy terephaldehyde followed by aptamer physical adsorption	EIS signal of the [Fe(CN)_6_]^3−/4−^ redox probe	Milk, river water, human serum, urine	31 fM–15.5 nM, 20 fM	[265]
5′-AGC AGC ACA GAG GTC AGA TGA CTT CAG TGA GTT GTC CCA CGG TCG GCG AGT CGG TGG TAG CCT ATG CGT GCT ACC GTG AA-3′	Au electrode modified with PDDA–graphene sheets and PtPd@Ni–Co MOF particles and covalently attached to capturing DNA	Aptamer–auxiliary DNA helix interacts with analyte and released auxiliary DNA into exonuclease III amplification cycle with hairpin DNA to form trigger DNA involved in the reaction with capturing DNA on the electrode interface. After that, signaling DNA bearing UiO-66 MOFs with MB molecules is attached and the MB signal measured in DPV mode	Honey	10 fM−10 nM, 0.985 fM	[266]
5′--SH-(CH_2_)_6_-AGC AGC ACA GAG GTC AGA TGC ACT CGG ACC CCA TTC TCC TTC CAT CCC TCA TCC GTC CAC CCT ATG CGT GCT ACC GTG AA-3′	FTO electrode modified with MoS_2_ nanoarray	Photocurrent measurements	Milk	0.1 pM–1 μM, 0.1 pM	[267]
5′-ACT TCA GTG AGT TGT CCC ACG GTC GGC GAG TCG GTG GTA G-3′	GCE modified with black phosphorus–Co-Ni/MOF–perylene derivative composite and physically adsorbed aptamer	ECL signal of persulfate cathodic reduction	Tap water	0.1 pM–1.0 μM, 0.29 fM	[268]

Abbreviations: COF—covalent organic framework, DPV—differential pulse voltammetry, ECL—electrochemical luminescence, Fc—ferrocene, FTO—fluorine-doped tin oxide, GCE—glassy carbon electrode, GO—graphene oxide, HRP—horseradish peroxidase, ITO—indium tin oxide, LO—limit of detection, LSV—linear scanning voltammetry, MB—methylene blue, MOF—metal–organic framework, MWCNTs—multiwalled carbon nanotubes, PDDA—poly(diallyldimethylammonium chloride), rGO—reduced graphene oxide, QD—quantum dot.

**Table 3:** Comparison of the general properties of the conventional methods of antibiotics detection with those based on electrochemical aptasensors.

Method	Specificity	Special Requirements	Detection Time	Cost
Microbiological	Low	Specialized bacteriological laboratory	Minimum 3 h	Modest
HPLC	High	Organic solvents	<1 h	High
GC	High	Sample should be transformed into gaseous phase	<1 h	High
MS	High	Sample should be transformed into ionized state	<1 h	High
MS–HPLC	High	Organic solvent, transformation of the sample into ionized state.	<1 h	High
MS–GC	High	Sample should be transformed into ionized state	<1 h	High
ELISA	High	Sandwich assay using expensive antibodies	<1 h	High
Electrochemical aptasensors	High	Antifouling surfaces for minimizing interferences with food matrix	<1 h	Low

## Data Availability

Not applicable.
